# GSP-AI: An AI-Powered Platform for Identifying Key Growth Stages and the Vegetative-to-Reproductive Transition in Wheat Using Trilateral Drone Imagery and Meteorological Data

**DOI:** 10.34133/plantphenomics.0255

**Published:** 2024-10-09

**Authors:** Liyan Shen, Guohui Ding, Robert Jackson, Mujahid Ali, Shuchen Liu, Arthur Mitchell, Yeyin Shi, Xuqi Lu, Jie Dai, Greg Deakin, Katherine Frels, Haiyan Cen, Yu-feng Ge, Ji Zhou

**Affiliations:** ^1^ College of Engineering, College of Agriculture, Academy for Advanced Interdisciplinary Studies, Plant Phenomics Research Centre, Nanjing Agricultural University, Nanjing 210095, China.; ^2^ Data Sciences, National Institute of Agricultural Botany (NIAB), Crop Science Centre (CSC), Cambridge CB3 0LE, UK.; ^3^ Department of Biological Systems Engineering, Department of Agronomy and Horticulture, University of Nebraska-Lincoln, Lincoln, NE 68583, USA.; ^4^ College of Biosystems Engineering and Food Science, Zhejiang University, Hangzhou 310058, China.

## Abstract

Wheat (*Triticum aestivum*) is one of the most important staple crops worldwide. To ensure its global supply, the timing and duration of its growth cycle needs to be closely monitored in the field so that necessary crop management activities can be arranged in a timely manner. Also, breeders and plant researchers need to evaluate growth stages (GSs) for tens of thousands of genotypes at the plot level, at different sites and across multiple seasons. These indicate the importance of providing a reliable and scalable toolkit to address the challenge so that the plot-level assessment of GS can be successfully conducted for different objectives in plant research. Here, we present a multimodal deep learning model called GSP-AI, capable of identifying key GSs and predicting the vegetative-to-reproductive transition (i.e., flowering days) in wheat based on drone-collected canopy images and multiseasonal climatic datasets. In the study, we first established an open Wheat Growth Stage Prediction (WGSP) dataset, consisting of 70,410 annotated images collected from 54 varieties cultivated in China, 109 in the United Kingdom, and 100 in the United States together with key climatic factors. Then, we built an effective learning architecture based on Res2Net and long short-term memory (LSTM) to learn canopy-level vision features and patterns of climatic changes between 2018 and 2021 growing seasons. Utilizing the model, we achieved an overall accuracy of 91.2% in identifying key GS and an average root mean square error (RMSE) of 5.6 d for forecasting the flowering days compared with manual scoring. We further tested and improved the GSP-AI model with high-resolution smartphone images collected in the 2021/2022 season in China, through which the accuracy of the model was enhanced to 93.4% for GS and RMSE reduced to 4.7 d for the flowering prediction. As a result, we believe that our work demonstrates a valuable advance to inform breeders and growers regarding the timing and duration of key plant growth and development phases at the plot level, facilitating them to conduct more effective crop selection and make agronomic decisions under complicated field conditions for wheat improvement.

## Introduction

As one of the most important staple crops in the world, wheat (*Triticum aestivum*) feeds over one-third of the world’s population, with its global consumption exceeding 790 million tonnes per annum in 2023 [1]. To ensure global food security for present and future generations, wheat production needs to be safeguarded against rapidly changing climates [2]. Hence, wheat breeders and growers need to regularly monitor key agronomic traits such as growth stages (GSs) (e.g., stem extension and flowering) and developmental phases (e.g., foundation, construction, and production) for crop improvement and agricultural production [3]. The plant’s essential growth cycle can be roughly divided into 10 stages [4], from germination (GS00-09) and seedling establishment (GS10-19) to grain filling (GS71-89) and ripening (GS91-99), among which critical stages such as tillering (GS20-29), stem extension (GS31-39), and flowering (GS61-69) are vital for yield formation and grain development. Hence, in recent years, much attention was paid to precisely monitoring and predicting the timing of these stages under field conditions, enabling breeders to select desirable genotypes from the plots and growers to arrange husbandry and agronomic inputs [5,6].

Plant GSs are sensitive to external stimuli in the field [7]. Different climatic factors can have varied effects on the plant, including (a) temperature, which could either delay or accelerate growth as different stages have specific temperature requirements [8]; (b) solar radiation and photoperiod, both of which can impact the speed of canopy development and influence the vegetative-to-reproductive transition (i.e., flowering) due to changes in the photosynthetic rate [9]; and (c) water availability (e.g., rainfall), which can lead to drought stress or waterlogging depending on whether the water supply is insufficient or excessive [10]. Traditionally, key stages were scored by field specialists based on vision features such as organic (e.g., leaves and spikes) and structural characters (canopy structure) together with climatic factors at different developmental phases [11]. This approach not only is laborious but also is subjective and lacks scalability, preventing breeders and researchers from performing large-scale evaluation of key stages from many lines in the plots, at different sites and across multiple seasons [6,12]. Moreover, manual scoring requires domain knowledge and experiences from individual breeders and researchers, which are not always available for all trial sites.

To alleviate these limitations, the application of biometeorological modeling to predict large-scale wheat phenological stages was first introduced to this research domain. Various agrometeorological factors such as temperature, photoperiod, rainfall, growing degree days (GDDs), and biometeorological time (BMT; i.e., interactions between the biosphere and the atmosphere across multiple seasons) were considered [13]. Examples of models used for forecasting wheat phenology and yields include thermal time models [14], the nonlinear multiplicative model [15], and ambient temperature enabled regression models [16]. Nevertheless, due to the complexity of field conditions and the short duration of some stages such as booting and heading (B&H; GS41-59) and flowering (GS61-69), biometeorological models struggled to precisely estimate short GS under a rapidly changing climate [17,18]. Moreover, many biometeorological models were primarily used for regional phenological stage predictions, which were not suitable for providing estimation for smaller regions (e.g., plots in the field, important for breeding and cultivation) due to their large-scale nature [16]. The above underlines the necessity of incorporating new techniques to improve toolkits for identifying plot-level GS [19].

Recently, remote sensing techniques combined with meteorological information were applied to wheat phenology studies [18]. The integration of moderate resolution imaging spectroradiometer (MODIS) data with phenological features was employed to facilitate the mapping of the winter wheat growth using satellite-equipped image sensors [20]. Time-series-based MODIS data were used to predict wheat maturity dates [21]. Still, satellite imaging has several disadvantages, including the revisiting periods (although recently improved, it still took days to revisit the same region) and relatively low-resolution images due to long distances, heavy cloud cover, and fog [22]. To identify key GS, other data collection methods such as aerial phenotyping and handheld devices have also been employed. For example, manned and unmanned aircrafts (e.g., drones) were applied to monitor crop growth and development at low altitudes [23]. Although popularly used in crop phenotyping, this approach has some limitations such as frequent changes in aviation regulations and different policies between countries [24], unsuitable weather conditions for drone flights [25], and fluctuating nature illuminance causing color and contrast distortions [26]. Ground-based equipment collected plant images in fixed or multiple positions, including Light Detection and Ranging (LiDAR) [27], handheld spectrometers [28], and selfie sticks with red-green-blue (RGB) cameras [29]. Similarly, these methods are limited in throughput, mobility, and scalability, restricting their use in large-scale and multisite crop surveillance.

Besides data collection, a variety of fitting functions and machine learning (ML) algorithms have been applied to assess growth patterns across different GS in cereal crops: (a) Time-series vegetation indices acquired by satellites were used to establish fitting curves with functions such as Gaussian and Savitzky–Golay filtering, enabling the identification of wheat varieties’ developmental profiles at the end of a season [30]; (b) support vector machine (SVM) and random forest (RF) were used to detect heading and ripening stages in rice based on canopy-level spectral and texture features [11]; (c) the compactness-separation principle algorithm was employed to recognize drone-collected vision features from wheat canopies, followed by the application of the multiclass correlation vector machine (mRVM) to classify heading and flowering stages [31]; (d) several fitting functions and RF-based classifiers were combined to monitor nitrogen responses after booting in winter wheat [32]; and (e) partial least squares regression was applied to predict rice heading dates using drone-collected multispectral and RGB images [33]. Many of these methods relied on feature engineering for ML modeling, incorporating specific textural, spectral, and color signals into the prediction. This required modelers to manually select and define biologically relevant features to fine-tune their models [34], which could decrease the generalization of the models due to insufficient domain knowledge, as well as unsuitable low-level (e.g., edges, textures, and colors) and high-level features (e.g., object parts and semantic concepts) [35]. Hence, the use of artificial intelligence (AI) and deep learning (DL) techniques is likely to help us learn these complex features through models with better predictive power to identify GS in wheat.

Through spontaneous feature learning in DL modeling, key input parameters can be learned by neural networks using multilayer nonlinear operations for generating desired features, which were proved to be able to obtain vision and semantic features from imagery [36]. For instance, convolutional neural networks (CNNs) were suitable for capturing both high- and low-level vision-based features from images [37], enabling DL models to learn canopy-level structural, spectral, and textural properties at different GS without excessive feature engineering. For patterns of climatic changes, previous studies employed recurrent neural network (RNN)-based LSTM networks [38,39], extracting temporal changes with thresholding mechanisms. This is valuable for integrating key climatic factors into the process of DL-based pattern recognition.

In order to address the challenges in identifying GS and predicting the vegetative-to-reproductive transition (i.e., flowering days) in wheat, we developed GSP-AI, a multimodal DL (MDL) model that combines time-series drone imagery with meteorological data for predictive modeling. The GSP-AI model has an efficient multiscale backbone architecture based on an optimized Res2Net-19 to extract color and textural features from wheat canopy images, along with an RNN-based LSTM network to capture patterns of climatic changes. To merge the 2 types of features (i.e., vision and climatic factors), a multilayer perceptron (MLP) was applied. To train the MDL model, an open Wheat Growth Stage Prediction (WGSP) dataset was established, consisting of 70,410 annotated canopy images and associated key climatic factors collected from 263 wheat varieties with 54 cultivated in China, 109 in the United Kingdom (UK), and 100 in the United States (US), among which 60,423 images were collected by drones from trilateral field trials (2018/2021 seasons) and 9,785 images were acquired by smartphones in the 2021/2022 season in China. After training, testing, and improvement, the GSP-AI model achieved a high accuracy for key GS identification and the flowering day prediction compared with manual scoring. Finally, to facilitate a broader community to access to our work, we developed several Jupyter notebooks [40] to integrate and execute the GSP-AI model, which are openly accessible through our GitHub repository together with the open WGSP dataset.

## Materials and Methods

### Plant materials and field experiments

**Fig. 1.**
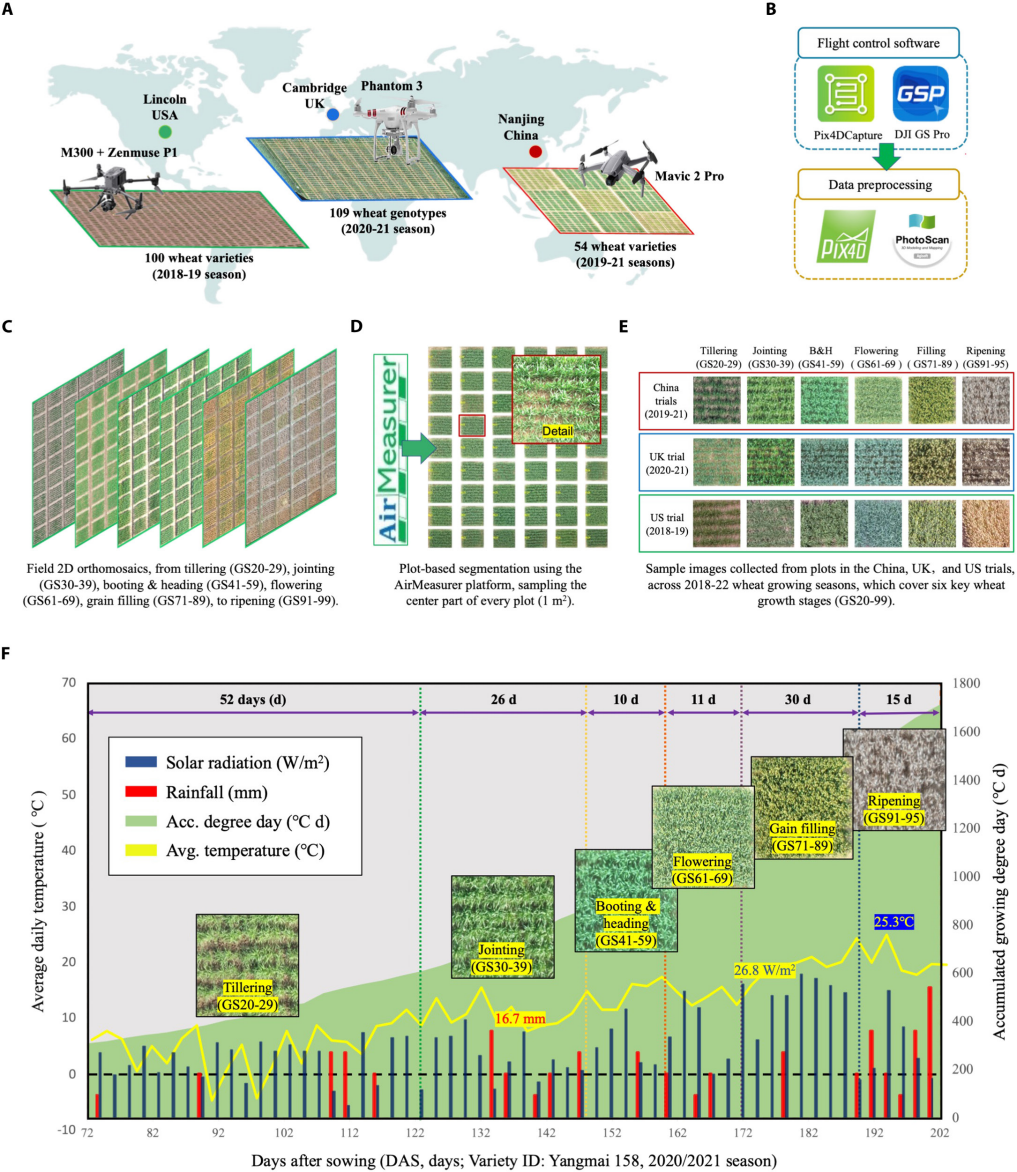
Drone phenotyping conducted in China, UK, and US to study 263 wheat varieties across 2018/2021 seasons, followed by the collection of canopy-level images and key meteorological data from tillering to ripening. (A) Three types of drones used to conduct drone-based aerial imaging of 263 wheat varieties cultivated in China (54 varieties), UK (109 varieties), and US (100 varieties) from 2018 to 2021 wheat growing seasons. (B) Trilateral aerial phenotyping standardized by similar flight control software and data preprocessing approaches. (C) A series of field-level 2D orthomosaics generated, covering 6 key GSs in wheat, from tillering, to jointing, B&H, flowering, grain filling, and ripening. (D and E) Plot segmentation accomplished by using the AirMeasurer platform, sampling the center part of every plot (around 1 m^2^ per plot) across the 3 countries. (F) Key GSs manually scored for a variety called YangMai 158 in the 2020/2021 season. Days after sowing (DAS) unit was used. Plot-based overhead aerial images are presented as references. GNSS information tagged in the images was associated with key climatic data such as solar radiation, rainfall, daily average temperatures, and accumulative growing temperature, which were obtained from weather stations and the International Weather Website (www.visualcrossing.com).

The 3 trial sites used for collecting wheat canopy images were across 3 continents and 4 growing seasons (Fig. 1A), including China (Asia), UK (western Europe), and US (North America). Based on drone-collected images, field-level 2-dimensional (2D) orthomosaics were first generated, based on which plot-level canopy images were obtained to build training and testing sets for DL modeling. Wheat varieties were cultivated for local geographic and climatic conditions (Table S1). In China, field experiments were established at the Nanjing Agricultural University’s Zhenjiang field trial center (NAU; 31°57′N, 119°18′E), across 2019/2021 seasons. Fifty-four varieties were examined, all of which were selected from the middle and lower reaches of the Yangtze River and other main wheat production regions in east China. Some of the varieties were known for dissimilar developmental rates [41]. The experiment contained 486 6-m^2^ (2 × 3 m) plots, with 25-cm row spacing and the sowing density kept 2.4 million plants per hectare (ha). Crops were drilled between 15th and 20th November in both seasons.

In the UK, the trial was conducted in the 2020/2021 season at the National Institute of Agricultural Botany’s (NIAB) Hinxton Big Common trial field (52°09′N, 0°18′E), with 109 genotypes selected from the chromosome segment substitution line (CSSL) population [42]. Plants were drilled in 240 12-m^2^ (2 × 6 m) plots on 2020 October 9, with a row spacing of 30 cm and a sowing density of 1.6 million plants per ha. In the US, the trial was based in Lincoln, Nebraska (96°70′N, 40°81′E). In the 2018–2019 season, 100 US prebreeding varieties were drilled in 100 12-m^2^ plots (2 × 6 m) on 17th October, with a row spacing of 30 cm and a sowing density of 2 million plants per ha. Across the 3 sites, all of the field experiments were suitable for both cultivation and breeding purposes, with one variety per plot. Plants were managed following standard husbandry practices, including appropriate agronomic inputs for water and fertilizers together with pest control according to local conditions.

### Drone-based phenotyping

Three types of drones were employed to perform the trilateral aerial phenotyping. In China, Mavic 2 Pro (DJI, Shenzhen, China) was used for 2 seasons and collected 26,023 raw images (5,472 × 3,648 pixels JPEG files, 298 GB in total). In the UK, Phantom 3 (DJI, Shenzhen, China) was employed to collect 8,010 images (4,000 × 3,000 pixels; 68 GB). In the US, DJI M300 equipped with a Zenmuse P1 camera (DJI, Shenzhen, China) was used to collect 6,124 images (8,192 × 5,460 pixels; 54 GB). We followed recommended practices previously described [43], including the installation of ground control points (GCPs) and height reference panels in the experiments. To largely standardize drone-based phenotyping, both DJI GS PRO and Pix4DCapture were used to perform aerial imaging to ensure image quality across different seasons, with similar imaging parameters such as speed, camera angle, and exposure mode (Fig. 1B and Note S1). To seek an optimal ground sampling distance (GSD) and thus an ideal image resolution for GS identification, 3 altitudes were applied in the drone phenotyping in the 3 countries: (a) at 14 m (GSD = 0.18 cm per pixel, cm·pixel^−1^; China), (b) 20 m (GSD = 0.25 cm·pixel^−1^; UK), and (c) 25 m (GSD = 0.32 cm·pixel^−1^; US). All of the drone flights were conducted 2 to 3 d before or after the manual scoring to maintain their relevance.

### Image preprocessing

Raw drone-collected images were first processed by the Pix4DMapper (Pix4D, Lausanne, Switzerland) and PhotoScan (rebranded to Metashape; Agisoft, St. Petersburg, Russia) software (Fig. 1B), generating calibrated 2D orthomosaics of the field experiments with global navigation satellite system (GNSS) information recorded from GCPs and reference points [44]. The collected aerial images ranged from tillering, jointing, B&H (combined due to their short timeframes), flowering, grain filling, to ripening, obtaining different color and textural properties of wheat canopies at different stages (Fig. 1C). Then, the AirMeasurer platform [44] was applied to automate the plot segmentation and remove plot edges, producing plot-level canopy images (around 1 m^2^ in size) for the following modeling activities (Fig. 1D). This resulted in a series of plot-based images (i.e., 4,212 images for China, 1,920 images for the UK, and 1,020 images for the US), which formed the core image set of the WGSP dataset (Table S2 and Fig. 1E).

### Ground-truthing of GSs and flowering days

Besides the canopy images, ground-truthing was conducted by field workers in the 3 countries. Field workers at the 3 sites scored GS according to the Zadoks decimal growth scale [4], including the timing and durations of a given stage, its progress, associated varieties, and daily meteorological data, all of which were cross-validated by other specialists either in the field or based on collected images. For the start of flowering or anthesis (GS61), due to different developmental paces of many varieties studied, the ground-truthing was largely obtained by the field workers’ observation together with estimation if flowering days were missed for some varieties (Tables S3 to S5).

### Image augmentation

Due to diverse timing and durations of GS in the trilateral trials, the wheat canopy images collected at different stages and in the 3 countries were marginally imbalanced (e.g., flowering images were 6 to 7% less than those collected at other stages; 2-season datasets collected in China were over 50% more than those in the UK and US due to an extra 2019/2020 season). Because the prediction accuracy of DL-based classifiers can be affected by imbalanced training set [45], we therefore applied image augmentation to enhance and balance the initial dataset. A variety of augmentation methods (e.g., luminance enhancement, rotation, and color adjustment) were chosen to mimic imaging-related problems that could be encountered in aerial field phenotyping, including changing natural illumination, out-of-focus photographs, and color distortion.

### Key climatic factors

As much previously published research did not fully incorporate climatic changes into predictive modeling [46,47], we first chose to use GNSS information tagged in the acquired images to associate meteorological data (Fig. 1F). Key climatic factors such as solar radiation (watts per square meter), rainfall (millimeters), daily average temperatures (°C), and accumulative GDD (also known as cumulative growing temperature; °C) were recorded from in-field weather stations and the International Weather Website (www.visualcrossing.com). These datasets enabled us to calculate seasonal accumulated solar radiation (ASR) and accumulation of daily effective temperature (ADET; Note S2). Based on standards previously defined [28], the base temperature (T_Base_) was set to 3 °C and the maximum effective temperature (T_Max_) was set to 30 °C for the Chinese trial, whereas T_Base_ was set to 0 °C and T_Max_ was set to 25 °C for the UK and US trials [48]. All the meteorological datasets used in this study are provided in Data S1.

### The learning architecture of GSP-AI model

**Fig. 2.**
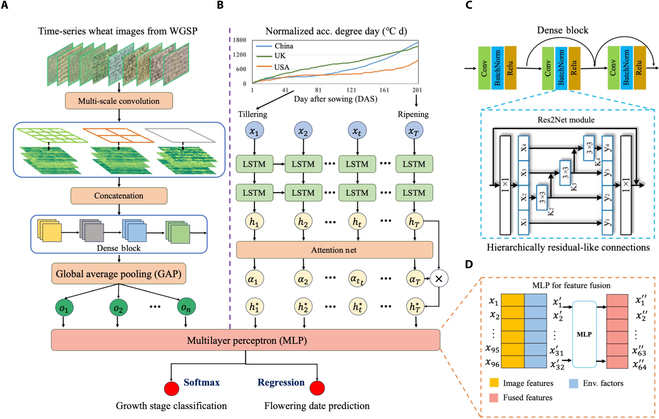
The learning architecture of the growth stage prediction (GSP) AI model (GSP-AI) used for predicting key GSs and flowering days in wheat. (A) The Res2Net backbone architecture used to learn vision-based features from wheat canopy images. (B) An RNN-based LSTM network was established to model climatic changes. (C) The residual blocks in ResNet were replaced by the Res2Net module together with customized Res2Net-19 used to extract multiscale features. (D) An MLP was applied to combine the 2-modal features from the 2 networks.

After establishing the training sets, we established the learning architecture for the GSP-AI model, which includes (a) the Res2Net backbone architecture [49] for vision-based feature extraction (Fig. 2A), (b) an RNN-based LSTM network [50] for modeling climatic changes (Fig. 2B), and (c) a multilayer perceptron to combine the 2-modal features from the 2 networks.

As different scales of wheat canopy features are crucial for GS detection, we first designed a multiscale receptive field to obtain multiple features from input images simultaneously. For example, to associate the above multiscale features, we chose the Res2Net module [51] to replace the residual blocks in ResNet and customized Res2Net-19 for feature extraction (Fig. 2C). This helped us obtain low-level features such as colors (e.g., changing from green to golden brown), corners, and edges (e.g., resulting in more textural features) from wheat canopies at different stages, including (a) the use of hierarchically residual-like connections within a single residual block to replace the traditional 3 × 3 convolutional kernel so that receptive fields at each layer could capture more fine-grained and diverse features from canopy-level leaves and spikes after booting; (b) the use of global average pooling (GAP) [52] instead of fully connected layers, preserving both spatial and high-level semantic information (i.e., an understanding of vision differences between GSs) while reducing network parameters and hence the model size; and (c) flattening aggregated features into a vector for extracting features.

To model the impacts of seasonal climatic changes at different GS, we selected the LSTM network (Fig. 2B), which is known for capturing the time-dependent relationship through the “gate” mechanism. In our case, key climatic factors (e.g., cumulative temperature and rainfall) and patterns of their changes were learned at every stage. Attention modules were applied to adjust the contribution of every hidden feature through normalized weights, consisting of a one-layer fully connected network with the Softmax function (see detailed modeling steps in Note S3). Features learned by the above 2 networks were combined by the feature fusion function. MLP was applied to fuse the 2-modal outputs from Res2Net-19 (i.e., a 96 dimension, D, image feature vector) and LSTM (i.e., a 32D climatic feature vector), including the following: (a) Both feature vectors were concatenated to form a fused feature vector with 128 dimensions; (b) MLP was created with a 128-neuron input layer to process the 128D feature vector, followed by a 128-neu hidden layer (HL1) with ReLU activation function [53], and a 64-neu hidden layer (HL2) to reduce the dimensionality of HL1’s output to 64; and (c) the output of HL2 formed a 64D fused feature vector (Fig. 2D) used for classifying key stages and predicting flowering days. The following equations (Eqs. 1 to 3) were used to perform the feature fusion:

$$F_{\mathrm{Img}}=f_{\mathrm{GAP}}\left(U\right)=\frac{1}{H\times W}\sum_{i=1}^{H}\sum_{j=1}^{W}U\left(i,j\right)$$ (1)

$$F_{\mathrm{Env}}=f_{\text{LSTM}}\left(\text{ADET},\mathrm{ASR},\text{Rain}\right)\;$$ (2)

$$F_{\text{Final}}=f_{\mathrm{MLP}}\left(F_{\mathrm{Img}},F_{\mathrm{Env}}\right)$$ (3)

where *H* and *W* denote the size of the feature map *U*, which is the global feature map (GFM) produced in the Res2Net; *F_Img_* and *F_Env_* denote the result vectors of vision-based features and climatic patterns, whereas *f_MLP_* stands for MLP to fuse features from both learning modes, with the final fused feature vector *F_Final_*; *f_LSTM_* stands for the LSTM model used to capture the time-dependent relationship, respectively. Additionally, *ADET* represents accumulation of daily effective temperature, *ASR* is accumulated solar radiation, and *Rain* stands for cumulative rainfall.

### Global feature maps

As feature maps (i.e., activation maps) play a crucial role in CNN-based architecture [54], we generated a range of feature maps to translate internal features from input images at different GS into visually recognizable patterns (i.e., GFMs). This helped us visually assess what types of features were learned by the AI model, based on which hyperparameters and key modules could be further improved. The GFMs were produced using the following steps: (a) The model generated feature maps through filters or feature detectors on the convolutional or pooling layers; (b) combining generated feature maps at different layers to create a global map, i.e., GFMs; (c) using the GFMs to visualize or quantify vision-based features (e.g., canopy and soil) based on their areas and important levels.

### Training strategy and model evaluation metrics

When training the GSP-AI model, the trilateral dataset was randomly divided into 70% (for training), 20% (for testing), and 10% (for validation) according to the total images collected from the 3 countries. Due to a high generality of low-level features (e.g., edges, corners, and colors) targeted at the canopy level across different GS, we therefore followed previously reported training strategy [55] and employed Transfer learning [56] to freeze the backbone features obtained through pretraining. With the back-end network retrained to update parameters in the AI model, we fine-tuned the top layers (i.e., fully connected layers) to achieve an optimal fitting for all the DL models examined (Fig. S1).

To train the 2-modal data and prevent overfitting, we chose Adaptive Moment Estimation (Adam) as the optimization algorithm with early stopping to compute an adaptive learning rate with a smaller memory requirement [57]. To compare prediction accuracies, we have included classical backbone architectures such as VGG-16, Inception-v3, DenseNet-121, ResNet-101, and ResNet-19 [58], whose architectures were different compared to GSP-AI. Input images were resized to 512 × 512 pixels and trained with parameters optimized for Res2Net (i.e., batch size = 8; gradient exponential decay rate, betas = 0.9; and learning rate = 0.0001). The time step of LSTM was set as 8 to secure that sufficient features from the present step could be transferred to the following step. The hidden unit was set as 100 to include adequate climatic changes within a season (variations between seasons were considered for the trial in China using factors such as accumulative GDD, ASR, and ADET).

Loss values (produced by the cross-entropy loss function for GS identification and the log-cosh loss function for flowering prediction) were employed as evaluation metrics [59], quantifying the deviation between predicted and annotated values (Fig. S1). To verify the performance of DL models, evaluation metrics such as accuracy, RMSE, and giga floating point operations per second (GFLOPS) were also used, along with the coefficient of determination (*R*^2^), RMSE, and mean error (ME) to validate classified GS and predicted flowering days against manually scored dates.

### Software implementation with Jupyter notebook

When training and implementing the GSP-AI model, a Windows 10 workstation (64 GB memory, Nvidia 2070 GPU, and Intel Core i7-8700 CPU) was used, along with TensorFlow (V2.2) framework [60] and Python (V3.8) for the model and software implementation. We applied the Exifread library (V3.0) to automatically obtain geographical coordinates from drone-acquired images [61]. Other key open scientific development libraries utilized in the study included the scientific data processing library SciPy [62] and the image processing library Scikit-Image [63]. The GSP-AI model was embedded in Jupyter notebook, which has been uploaded to our GitHub repository with detailed runtime environment and system configuration guidelines provided (see the “Open Access” section).

## Results

### An open WGSP dataset

**Fig. 3.**
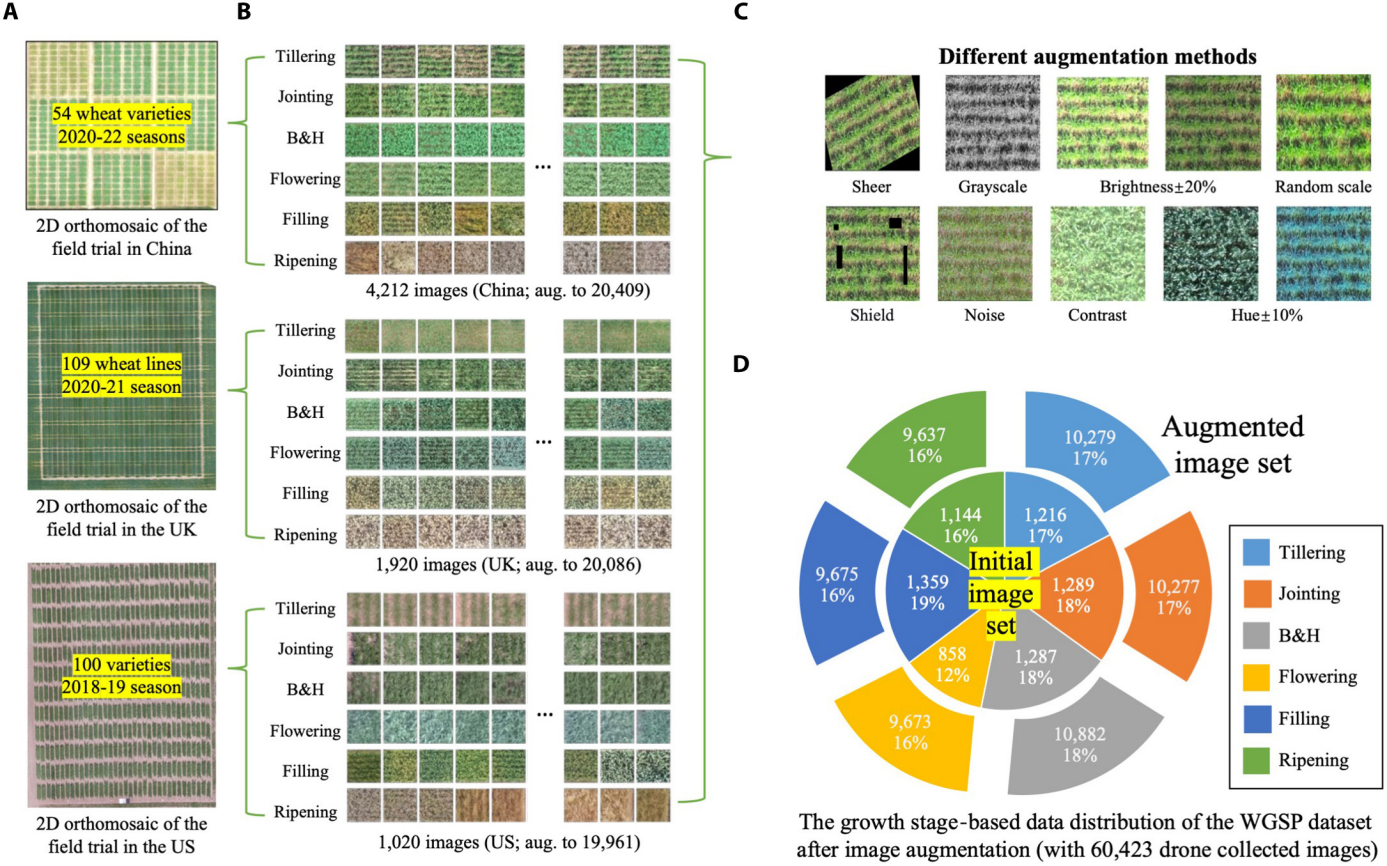
The open WGSP dataset established using drone-collected canopy images for training, testing, and validating the GSP-AI model. (A) 2D orthomosaics generated using raw images collected by drones from the trilateral field trials. (B) Examples of plot-level canopy images collected at 6 key GSs with the initial and augmented numbers of images per country. (C) Examples of different image augmentation methods used. (D) A pie chart showing the number of initial images on the inner circle and the number of augmented images on the outer pie segments, demonstrating the data distribution of WGSP at every GSs.

Building on the trilateral canopy images acquired by drones from the 263 varieties in China, the UK, and the US (Fig. 3A), we first established an open WGSP dataset. Because the 263 lines possessed dissimilar developmental rates and various canopy color/structural features, the initial WGSP dataset comprises images at 6 key stages (inner pie segments; Fig. 3D) phenotyped at 3 GSDs (i.e., 0.18, 0.25, or 0.32 cm·pixel^−1^), from tillering (1,216 images), jointing (1,289 images), B&H (1,287 images), flowering (858 images), grain filling (1,359 images), to ripening (1,144 images). To balance the number of images at every GS (Fig. 3B), different image augmentation methods were applied (Fig. 3C). This resulted in an augmented dataset of 60,423 canopy images at approximately 10,000 images per GS (outer pie segments; Fig. 3D) and around 20,000 images per country: tillering (10,279 images), jointing (10,277 images), B&H (10,882 images), flowering (9,673 images), gain filling (9,675 images), and ripening (9,637 images).

In order to further improve the generalization of the GSP-AI model, we added unseen wheat canopy images collected by Android smartphones (3,968 × 2,976 pixels) in a Chinese case study during the 2021/2022 season. With overhead cameras maintained 2 m away from wheat canopies (i.e., a higher GSD of 0.05 cm·pixel^−1^), the augmented image set consists of 9,987 images, including tillering (1,655 images), jointing (1,652 images), B&H (1,643 images), flowering (1,657 images), gain filling (1,692 images), and ripening (1,688 images), which were used to increase the WGSP dataset to 70,410 annotated images. The GeoSetter software [64] was employed to tag GNSS information with annotated images in the WGSP, dependent on which key climatic factors were retrieved from the International Weather Website (see key climate factors in Fig. S2).

### The GSP-AI model trained with drone images and climatic factors

In order to yield better prediction accuracy, we first used the WGSP dataset to train 6 learning architectures as backbone networks of the GSP-AI model, including VGG-16, Inception-v3, DenseNet-121, ResNet-101, ResNet-19, and Res2Net-19. After fine-tuning network hyperparameters to obtain an optimized prediction accuracy (Fig. S1), we found that Res2Net-19 performed the best in GSP (with an average accuracy of 85.4%) and RMSE (<7 d) for predicting flowering days (Table 1). Hence, we chose Res2Net-19 as the backbone network to characterize different scales of vision features at the canopy level. The model used a GAP layer to reduce network parameters and computational complexity, helping the model’s future deployment. To improve the prediction accuracy, key climatic factors and their patterns were integrated into the model through the LSTM network (Fig. 2B). We combined outputs from LSTM and Res2Net-19 with an MLP. After retraining the Res2Net-19 + LSTM model, the final model was 10.4 MB in size (significantly smaller than other models), with an overall accuracy of 91.2% for identifying GS (5.8 to 10.3% higher than other models) and an average RMSE of 5.6 d across all stages for predicting flowering days (Table 1).

**Table 1.** Comparisons of the trained DL models with the GSP-AI model using various evaluation metrics

Model name	Overall GSP accuracy	RMSE of flowering days	Parameters (million)	GFLOPS (1 billion per second)	Model size (MB)
VGG-16	80.9%	13.2 d	138	134.9	480
Inception-v3	82.1%	12.1 d	27.7	85.8	157
DenseNet-121	81.9%	9.9 d	12.1	27.9	28
ResNet-101	82.9%	8.6 d	52.7	44.5	163
ResNet-19	83.1%	7.8 d	21.4	31.2	44
Res2Net-19	85.4%	6.9 d	5.6	12.4	9.8
Res2Net19+LSTM (GSP-AI)	91.2%	5.6 d	6.1	20.1	10.4

### Improved prediction accuracy with GFMs and key climatic factors

**Fig. 4.**
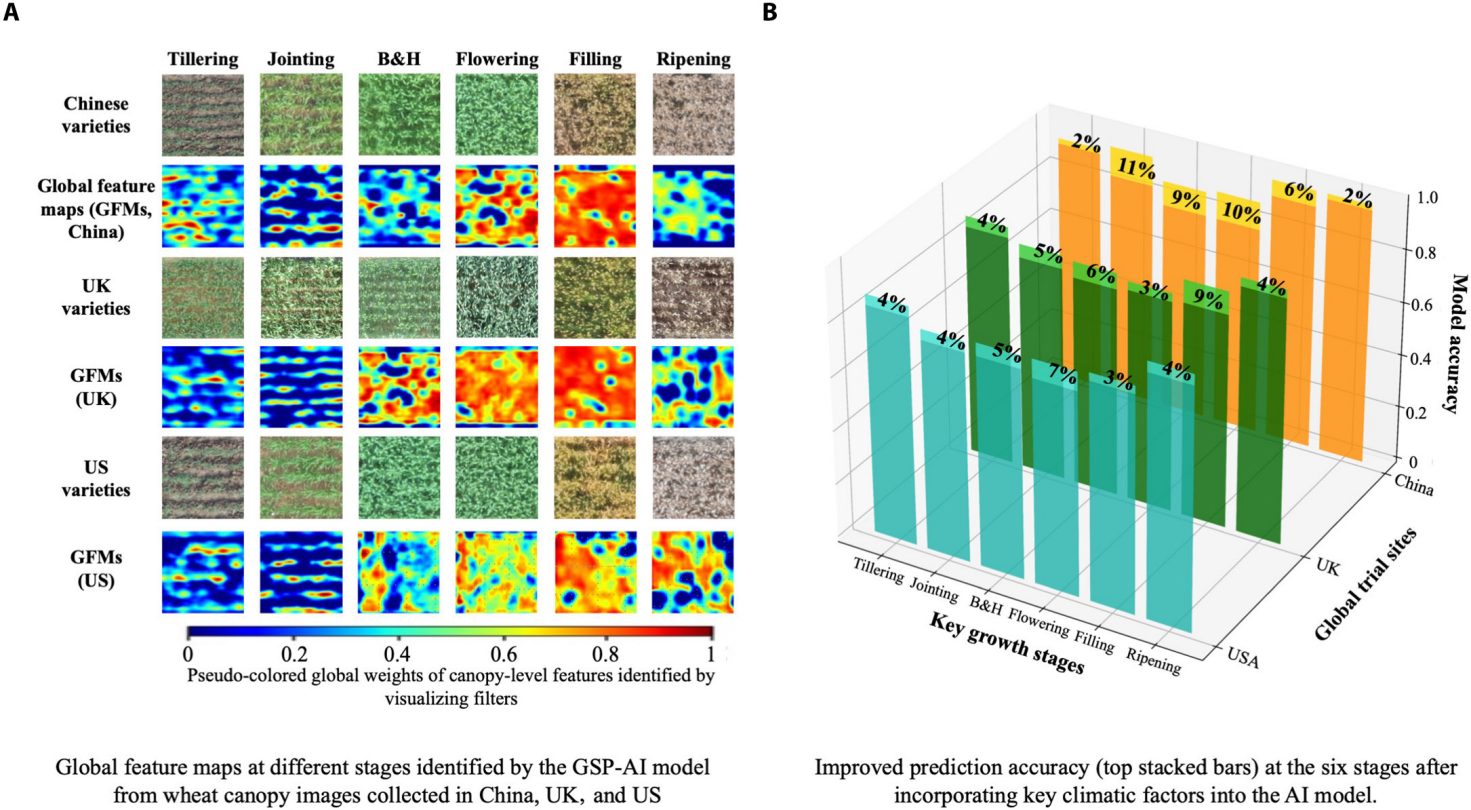
GFMs developed to visualize vision features or patterns at 6 GSs, along with improved prediction accuracy after incorporating key climatic factors. (A) GFMs produced at the 6 stages using the GSP-AI model identified key features learned by the AI model from wheat canopy images collected in China, UK, and US. (B) Improved prediction accuracy (top stacked bars) at 6 stages after incorporating climatic factors into the GSP-AI model.

As CNN-based models use filters to convolve feature maps learned from the convolutional layers, weights and bias values from the layers can be exploited to adjust hyperparameters [65]. In our case, we combined intermediate representations of wheat canopy images across different GS to generate GFMs, which detected diverse vision-based features or patterns learned by the GSP-AI model (Fig. 4A). Using stage-based GFMs, we assessed whether extracted canopy-level features and regions were compatible with field specialists’ visual evaluation, based on that we fine-tuned hyperparameters and learning architecture (e.g., bottleneck block, kernel length, scale dimension, and global feature fusion) to improve the GS identification. This helped us achieve an optimized prediction accuracy through maximizing target features such as the plot-level plant regions for key organic features (e.g., leaves and spikes) and their significant levels against those derived from background signals (e.g., soils).

After that, key climatic factors were integrated into the GSP-AI model, showing an overall improvement in prediction accuracy (5.8% higher averagely) compared with the results without considering climatic factors (Table 1 and Fig. 4B). Prediction accuracies based on China, UK, and US images were improved by 6.8% (overall 93.1%, across all 6 stages), 4.9% (overall 87.4%), and 4.4% (overall 85.8%), respectively (Table S6), indicating usefulness to incorporate key climatic factors into the GSP-AI model. Additionally, it is noticeable that the improvements after integrating key climatic factors were achieved largely at stages with relatively short durations (e.g., jointing, B&H, and flowering, with an average increase of over 6%), whereas the increase of prediction accuracies of tillering and ripening stages was marginal (Table S6).

### The performance of GSP-AI in predicting flowering days

**Fig. 5.**
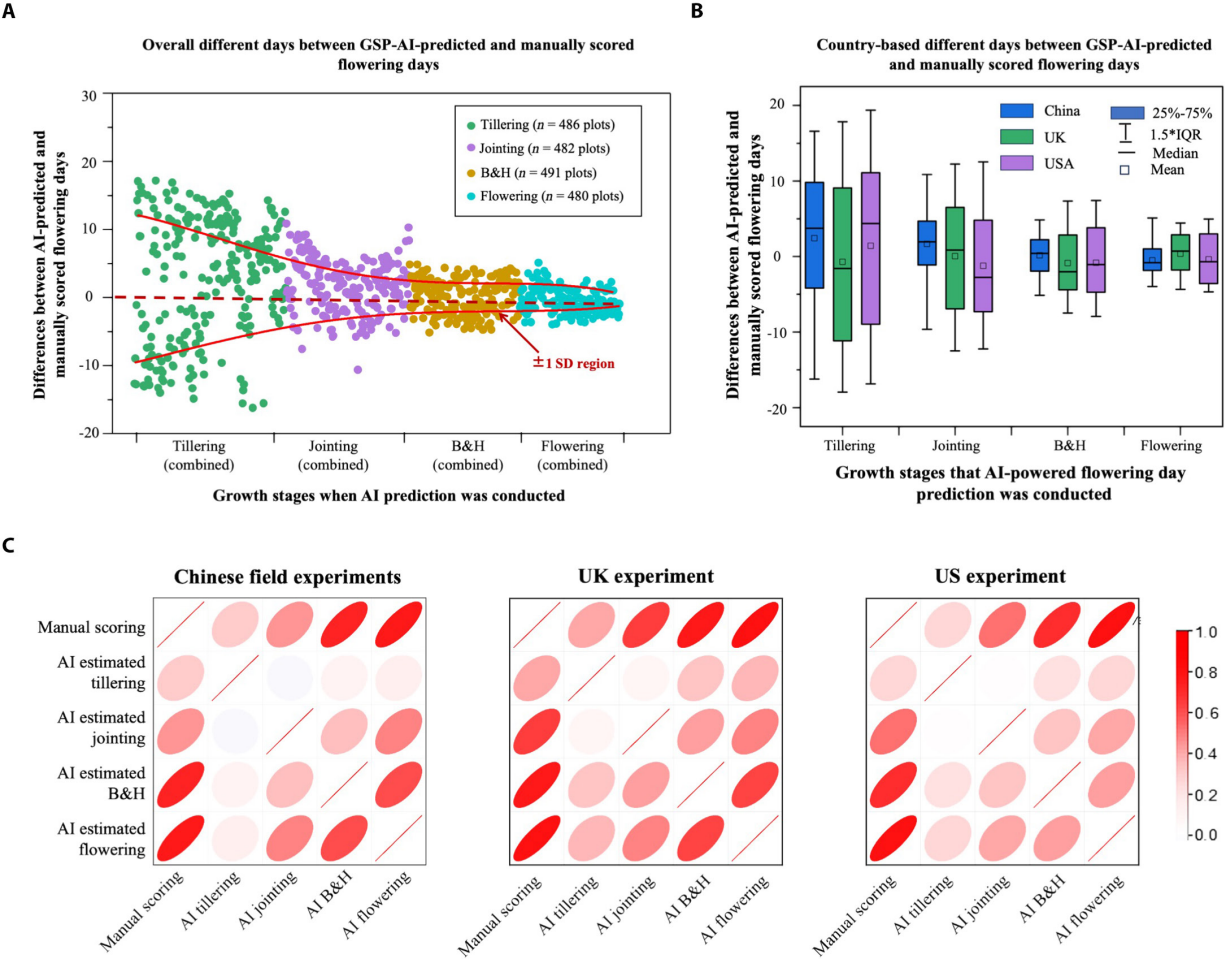
The performance of flowering day prediction made by the GSP-AI model with correlation analyses between manual scoring and AI-predicted results. (A) Overall differences between GSP-AI-predicted and manually estimated flowering days at different GSs. (B) Differences between GSP-AI-predicted and manually scored flowering days for every country, with error ranges, mean, and medium values of the prediction days provided. (C) Three correlation matrices produced to show the results of Pearson correlation analysis at 4 GSs. Variations (i.e., narrower denoting smaller variations) and directions marked with different shades of colors, from white to red (0 to 1) for positive correlations on the red-color scale (no negative correlations were observed).

We further validated the prediction of flowering days made by the GSP-AI model using RMSE, resulting in an average accuracy of 5.6 d across tillering, jointing, B&H, and flowering stages (note: the flowering stage was included in the prediction due to diverse developmental paces across the 263 varieties), compared with 9.75 d made by the other backbone networks (Table 1). Nevertheless, when dividing the flowering prediction made at different stages, it is evident that the prediction at tillering had the largest deviation (ME = −10.4 to 12.1 d; i.e., prediction was either delayed by 10.4 or 12.1 d before of the manual scoring). The closer to the flowering days, the more accurate the predictions became (Fig. 5A; standard deviations highlighted with 2 red lines). The RMSE of flowering prediction at jointing stage was 4.9 d (ME = −6.1 to 6.7 d), whereas MEs at both B&H and flowering stages were −3.9 to 4.1 d and −2.4 to 2.7 d, respectively (Tables S3 to S5).

Additionally, we compared the stage-wise variability in the flowering prediction across the 3 countries. The AI-powered flowering prediction based on Chinese images had the smallest variance (ME = −4.7 to 9.9 d, −2.1 to 4.8 d, −2.6 to 2.5 d, and −2.3 to 1.3 d across the 4 stages), whereas the variances in the UK and US trials were rather similar. This was further compared using the correlation analysis of manually scored and predicted flowering days (Fig. 5C and Table 2; *P* < 0.001), demonstrating a tendency of improvement in AI-powered prediction when estimations were made close to the flowering stage. At B&H and flowering, average *R*^2^ values for the analyses were 0.76, 0.80, and 0.76 for China, UK, and US trials, respectively (average RMSE = 2.3, 3.4, and 4.1 d).

**Table 2.** *R*^2^ and RMSE values calculated to evaluate correlations between AI-predicted flowering days and manual scoring in the trilateral field experiments

Key stages	*R*^2^ (China)	RMSE (China)	*R*^2^ (UK)	RMSE (UK)	*R*^2^ (US)	RMSE (US)
Tillering	0.306	9.1 d	0.392	10.6 d	0.31	10.8 d
Jointing	0.448	4.3 d	0.61	7.6 d	0.451	7.5 d
B&H	0.723	2.6 d	0.781	4.2 d	0.731	5 d
Flowering	0.794	1.9 d	0.813	2.6 d	0.786	3.1 d

### Verify and improve the GSP-AI model through a case study

**Fig. 6.**
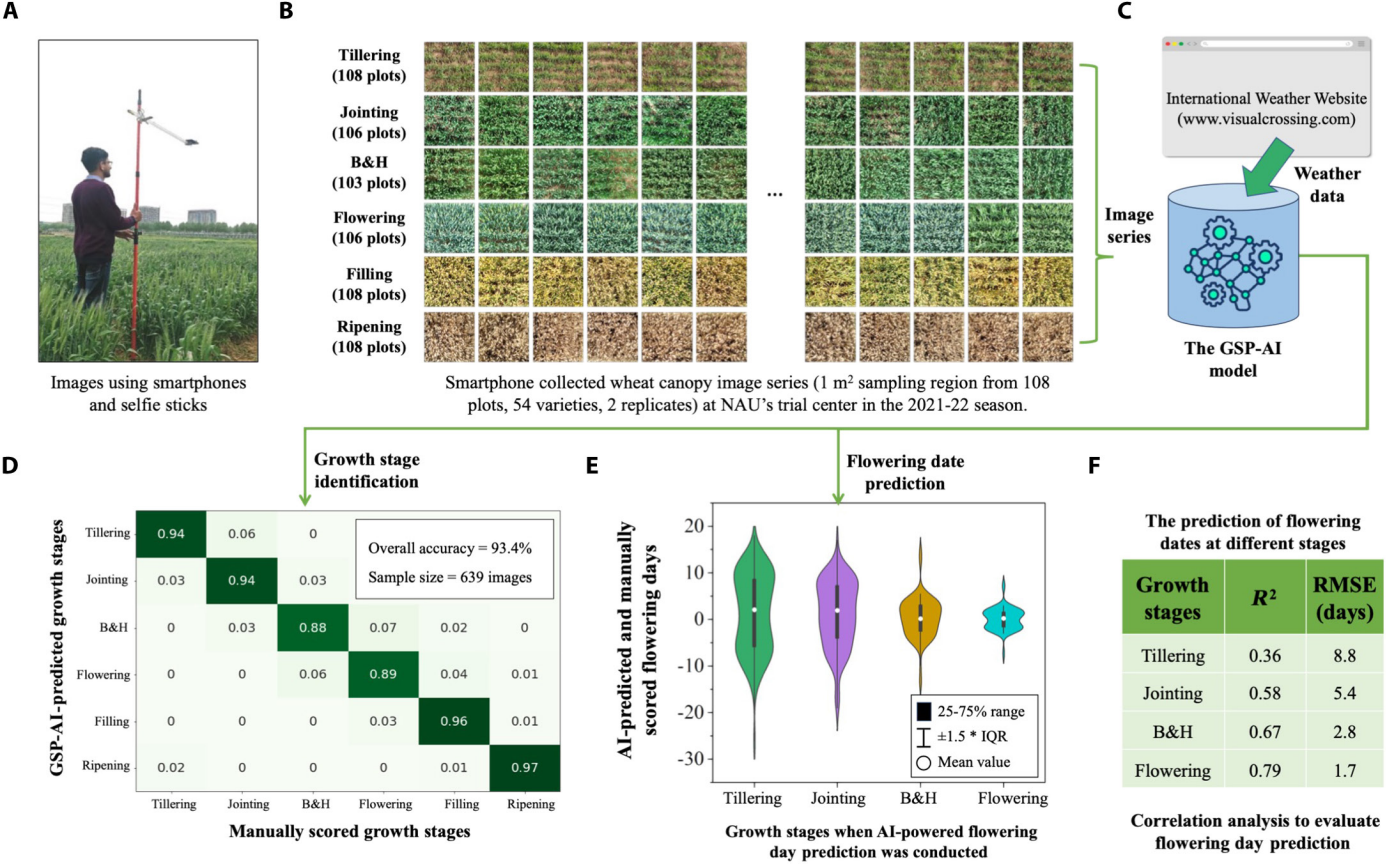
Smartphone-collected images were used to verify and improve the GSP-AI model in a case study in China, resulting in an improved accuracy of GS identification and flowering day predictions. (A and B) An adjustable imaging stick mounted Android smartphones used to collect a series of wheat canopy images from 108 plots, at 6 key GSs. The central part of every image (about 1 m^2^ in size) was sampled. After image augmentation, 9,987 images were obtained and added to the WGSP dataset. (C) Key climatic factors associated with smartphone-collected images. (D) A confusion matrix produced to show results for images collected at tillering, jointing, filling, and ripening stages, resulting in 94 to 97% identification accuracy. (E and F) A violin diagram produced to display deviations of the predicted flowering days made at 4 stages compared with manual scoring, followed by the correlation analysis using *R*^2^ and RMSE to evaluate the accuracy of predicted flowering days.

In the 2021/2022 season, we applied the GSP-AI model to identify GSs using unseen and high-resolution smartphone images to verify and improve the GSP-AI model. through an in-field case study. After establishing a field experiment in Nanjing China with 54 winter varieties (Table S1), we first used adjustable imaging sticks to mount Android smartphones to collect a series of wheat canopy images from 108 plots (1.75 × 3 m) at 6 key stages (Fig. 6A). The central part of every plot (about 1 m^2^ in size) was sampled (Fig. 6B), followed by the application of the GSP-AI model to identify GS based on these images together with climatic factors obtained from the International Weather Website (Fig. 6C). In total, 9,987 images were produced after image augmentation, which were largely evenly distributed across the 6 stages (approximately 1,660 per stage; Table S7). For GSP, a confusion matrix (Fig. 6D) was created to show that the prediction accuracies for tillering, jointing, filling, and ripening stages were 94 to 97%. Because prediction accuracies of B&H and flowering stages were 88% and 89%, we further improved the model by fine-tuning the hyperparameters in the architecture (e.g., changing kernel length and scale dimension). Eventually, we achieved an overall accuracy of 93.4% for GS identification, slightly higher than those made based on drone-collected images.

After that, we used the improved model to predict flowering days. A violin diagram was produced to display deviations of the predicted flowering days made at 4 stages compared with manual scoring (Fig. 6E). Similar to the predictions made on drone images, the diagram shows that the deviation was greater at tillering (ME = −9.4 to 11.9 d) compared with other stages. The closer to flowering, the more converged the predictions were (ME = −2.3 to 1.9 d). The overall prediction results (4.7 d) were slightly better than the GSP-AI-predicted days based on drone images. We further computed *R*^2^ and RMSE to evaluate the accuracy of predicted flowering days made from tillering to flowering, resulting in *R*^2^ = 0.36 to 0.79 (Fig. S3) and RMSE = 8.8 to 1.7 d (Fig. 6F). Similar to the prediction made on drone-collected images, the AI-predicted flowering days made at B&H and flowering using smartphone images were just 2.3 d (*R*^2^ = 0.73) away from the manual scoring, indicating its biological relevance.

## Discussion

AI-powered techniques are suitable for identifying patterns, making predictions, and deriving explicit and implicit relationships from big multidimensional datasets [66], offering breeders and plant researchers transformative solutions to gain biological insights that are capable of addressing global challenges of food security, environmental sustainability, and climate-resilient agriculture [67]. In this study, we demonstrate how AI-powered DL modeling was employed to identify GS and predicting flowering days using wheat canopy images and key climatic factors. To our knowledge, reliable and scalable toolkits that combine vision features with climatic patterns to estimate plot-level timing and durations of key GS in wheat are still not available, indicating valuable advances made by our work.

### WGSP—An open dataset for identifying key GSs in wheat

DL and ML techniques require high-quality training data to facilitate model training and tuning, which has become a bottleneck that has prevented plant and crop research community from fully exploiting AI-powered technologies in recent years [68]. The Wheat Head Detection Dataset (GWHD) [69], Diverse Rice Panicle Detection (DRPD) [36], and Plant Village [70] datasets contained hundreds of thousands of labeled images with wheat spikes, rice panicles, and plant disease symptoms, providing diverse and training-ready annotated data to accelerate crop yield and plant pathological studies. Following this trend, we also established the open WGSP dataset to share annotated GS-related wheat canopy images with the community. The WGSP dataset was built upon thousands of raw images collected by drones (2018/2021 seasons) and smartphones (2021/2022 season) from trilateral field experiments. It consists of 70,410 annotated plot-level canopy images sampled from wheat canopies together with key climatic factors, covering key vegetative and reproductive phases during wheat growth cycles. The data distribution in the WGSP dataset is (a) approximately 11,700 images per stage, (b) around 30,000 images for the Chinese varieties, and (c) around 20,000 images for the UK and US trials. By developing this openly accessible dataset, we trust that it will help plant researchers and breeders continuously develop AI-powered predictions of GS and flowering days, advancing collaboration and transparency in data-driven research, which is likely to render a wide adoption of the WGSP for AI-powered plot-level predictive modeling for breeding and agronomy.

### Capturing multiscale vision features from crop canopies

The term “black box” is often used to describe DL modeling due to the lack of interpretability in the decision-making processes conducted by DL models. This was mainly caused by the complexity of deep neural networks, high-dimensional spaces of input datasets, nonlinearity relationships derived from AI prediction, and hierarchical features that were impractical to explain the derived rules [71]. To understand key vision-based features that should be learned by the AI model at different stages, we worked with field workers to incorporate their domain knowledge, including (a) expected plot color changes during wheat growth and development, (b) canopy-level textural differences when flag leaves unfolded and spikes emerged at B&H (GS41-59), (c) canopy color gradually turned into light pale green with texture becoming nonhomogeneous at flowering (GS61-69) due to visible spikes and anther extrusion, and (d) when plant color changed to light brown and then golden brown due to grain development and dehydration at grain filling and ripening (GS71-89).

To extract and compare the fine-grained features, we chose Res2Net-19 as the backbone network due to its strong ability to represent multiscale vision features [34]. Res2Net-19’s receptive fields included different scales of boundaries, regions, and semantic categories of target objects (e.g., canopy texture, color, and other morphological features), which learned both low- and high-level features from the wheat canopy. Through this approach, stage-based GFMs were produced, representing blue-colored regions (i.e., lower weights identified by filters) for soil signals and red-colored regions (i.e., higher weights and thus higher attentions) for canopy-level plant signals. The GFMs helped us and field workers jointly assess and verify key areas highlighted at different stages, which led to the improvement of hyperparameters and the learning architecture to match highlighted regions with field workers’ attention areas. Moreover, we were able to align areas with high weights in the GFMs with the annotated images before and during flowering, enabling us to study key vision differences for flowering.

### Incorporating climatic changes into the AI model

Besides vision features, we also incorporated an RNN-based LSTM into the GSP-AI model to learn sequential climatic change patterns over time. As LSTM can handle time-series measures of temperature, solar radiation, and rainfall, we therefore employed the network to integrate seasonal variations and weather patterns into the predictive model. As wheat plants need to accumulate a certain level of solar radiation, nutrients, and water before progressing to the next phase [72], we included climatic factors (e.g., cumulative growing temperature, solar radiation, and rainfall) in the prediction of GS, which improved prediction accuracies of China, UK, and US varieties by 6.8% (93.1% across all 6 stages), 4.9% (87.4%) and 4.4% (85.8%), respectively. Furthermore, this approach noticeably improved the prediction accuracy of flowering days, which are extremely sensitive to external stimuli [73]. We observed an average increase of 18.8% in RMSE values (i.e., 3.3 d compared with manual scoring), when the prediction was made from B&H onward. When investigating the prediction at 4 key stages, significant increases were observed, from tillering (*R*^2^ = 0.336, RMSE = 10.17 d) to flowering (*R*^2^ = 0.798, RMSE = 2.53 d), with an improvement of 137.4% (in *R*^2^) and 75.1% (for RMSE). The above results can be continuously improved when more environmental factors (e.g., soil conditions), wheat genetic characteristics, and agronomic management (i.e., G × E × M) are incorporated into the GSP-AI model, which will be valuable to trial in future studies of flowering prediction.

### Limitations of the study and future development

It is noticeable that the prediction accuracies between different regions and stages varied to some extent (e.g., the results based on datasets collected in China was 5 to 10% better in terms of GS identification and 1 to 2 d better in flowering day prediction). We believe that the reasons are as follows: (a) data quality: the GSDs of canopy images in China were 0.18 cm·pixel^−1^ for drone phenotyping and 0.05 cm·pixel^−1^ for smartphone imaging in comparison with 0.25 cm·pixel^−1^ (UK) and 0.32 cm·pixel^−1^ (US); better image resolutions likely retain more low- and high-level vision features, which can positively impact the AI model’s performance; (b) data distribution: although we used the image augmentation method to balance feature and class distribution, one season trial in the US and UK was likely affecting overall accuracy due to possibly unrepresented features at different site and dissimilar GS in the training data; (c) dataset size: a smaller image and climatic dataset in the US and UK may not provide sufficient examples for the AI model to learn canopy-level vision features and climatic patterns between multiple seasons and stages effectively, leading to relatively lower accuracy and increase after incorporating the climate factors; (d) data complexity: we only used key climatic factors without soil and agronomy information, which might have underestimated the complexity of the patterns of G × E × M, and thus, the model was not able to capture such features. In summary, the variability in prediction accuracies across different countries and GS was likely influenced by the quality and characteristics of the annotated datasets, which could be improved due to the open-source nature of the WGSP dataset.

While the results of this study were promising, further improvements still need to be considered, including (a) the addition of agronomic activities such as cropping conditions in every trial location together with detailed comparative analysis of the prevailing weather conditions; (b) the integration of existing regional biometeorological models used for predicting phenological stages in wheat, which can potentially bring different factors to the AI modeling; (c) while wheat varieties selected in the study were representative lines in the 3 countries, a more diverse set of genotypes with varied characteristics of growth cycles and canopy-level morphological and physiological features shall be included with multiseasonal and multisite trials; (d) flowering days and its duration can be collected and cross-referenced by different teams across different trial sites, with performance metrics such as the Nash–Sutcliffe efficiency coefficient [74] applied to compare and improve the manual scoring.

Besides experimental and validation improvements, the WGSP dataset and the GSP-AI model can also be improved by including images between GS to include the transition phases of wheat growth and varieties with more diverse developmental paces cultivated in other wheat production regions. Wheat canopy images with dissimilar GSDs, growth patterns, and imaging sources (e.g., multi- and hyperspectral image sensors) are likely to enrich the WGSP dataset and thus increase multiscale dynamic representations of wheat canopies during the season for the GSP-AI model to examine. Annotations with metadata descriptors to provide detailed plot-level information such as varietal name, experimental conditions, positional information, sampling methods, and other relevant data sources such as micro-climatic changes at the field level can be included to map the semantics of GS-related data with vision, spatial, and temporal changes in an embedding space, leading to standardized and interpretable DL modeling.

AI-powered analysis requires community engagement and regular feedback. To foster engagement and collaboration with end users (e.g., breeders and plant researchers) and developers (e.g., AI and data scientists) for plant and crop research, joint contributions and online forums are needed to ensure the relevance, accuracy, and reusability of the work presented here. From an AI implementation’s perspective, Res2Net-19 and LSTM performed well in capturing fine-grained canopy features and temporal climatic changes from the input datasets. Still, these phenotypes were not collected under biotic or abiotic stresses, which could also dramatically affect the timing and duration of GS. Hence, future developments could also consider data collected under varied external stimuli together with the latest multihead attention mechanism such as Transformers [75], extracting new high-level features from multimodal data and helping the GSP-AI model to provide a more accurate and generalized prediction of GS and flowering days.

## Conclusion

The multimodal GSP-AI model introduced here combines drone-collected drone imagery and key climatic factors to accurately identify key stages and predict flowering days in wheat plots. To build the AI model, we utilized both Res2Net-19 and LSTM to obtain canopy-level vision features and patterns of climatic change, followed by MLP to fuse different features for predictions. We trained the AI model with the open WGSP dataset, which consists of 70,410 images collected from 263 wheat varieties cultivated in China, UK, and US from trilateral and multiseasonal field trials. After verifying and improving the AI model with the cross-country trials and unseen data in a case study using smartphone-based images, we reached an overall accuracy of 93.4% for GS identification and RMSE of 4.7 d for the flowering prediction. To our knowledge, such an AI-powered approach for identifying GS and predicting flowering days at the plot level for wheat plants has not yet been introduced. Hence, we trust that our work is likely to provide breeders, plant researchers, and growers with a new toolkit that leverages the power of AI algorithms to optimize variety selection strategies, crop monitoring, and agronomic management decisions making to sustain crop performance in the field.

## Data Availability

Source code and algorithm of GSP-AI are distributed under the Creative Commons Attribution 4.0 international license, permitting academic use, distribution, reproduction in any medium, provided you give appropriate credit to the original authors and the source, provide a link to the Creative Commons license, and indicate if changes were made. Unless otherwise stated, the Creative Commons Public Domain Dedication (http://creativecommons.org/licenses/by/4.0) waiver applies to the data and results made available in this paper. Source code, WGSP, and other datasets supporting the results presented in this article are available at https://Github.com/The-Zhou-Lab/GSP-AI/releases. Other source code, data, and user guides are openly available on request. The AirMeasurer platform used for plot segmentation and canopy sampling can be accessed via https://github.com/The-Zhou-Lab/UAV/releases.

## Supplementary Materials

Supplementary 1 Notes S1 to S3 Figs. S1 to S3 Tables S1 to S7 Data S1 References
