# Supplementary material for: GSP-AI: An AI-Powered Platform for Identifying Key Growth Stages and the Vegetative-to-Reproductive Transition in Wheat Using Trilateral Drone Imagery and Meteorological Data
Source: Plant Phenomics. 2024 Oct 9;6:0255. doi: 10.34133/plantphenomics.0255 (PMC11462051; doi:10.34133/plantphenomics.0255)
Supplement: Supplementary 1 — Notes S1 to S3 Figs. S1 to S3 Tables S1 to S7 Data S1 References [file plantphenomics.0255.f1.docx]

**Supplementary Material**

## **Title:** GSP-AI: An AI-powered platform for identifying key growth stages and the vegetative-to-reproductive transition in wheat based on trilateral and multi-seasonal drone phenotyping

**Authors:** Liyan Shen^+^, Robert Jackson^+^, Guohui Ding^+^, Shuchen Liu, Mujahid Ali, Arthur Mitchell, Yeyin Shi, Xuqi Lu, Jie Dai, Greg Deakin, Katherine Frels, Haiyan Cen, Yu-feng Ge, Ji Zhou*

^+^ Contributed equally; ^*^ Corresponding authors

**Table S1.** The full list of China/UK/US wheat varieties studied in the field experiments.

| **ID** | **China varieties (n = 54)** | **UK varieties (n = 109)** | **US varieties (n = 100)** |
| --- | --- | --- | --- |
| 1 | Xumai32 | CSSL.1A.01 | UNL460 |
| 2 | Dongmai1301 | CSSL.1A.02 | UNL104 |
| 3 | Weilai1216 | CSSL.1A.03 | TAM 113 |
| 4 | Wanmai52 | CSSL.1A.04 | WB GRAINFIELD |
| 5 | Wanma108 | CSSL.1A.05 | UNL356 |
| 6 | Wanyu2 | CSSL.1A.06 | TAM 304 |
| 7 | Tianmin108 | CSSL.1B.01 | UNL56 |
| 8 | Pingmai108 | CSSL.1B.02 | Freeman |
| 9 | Lvyu098 | CSSL.1B.03 | UNL118 |
| 10 | Kenong9204 | CSSL.1B.04 | UNL46 |
| 11 | Baoji0601 | CSSL.2A.01 | UNL7 |
| 12 | Aikang58 | CSSL.2A.02 | UNL161 |
| 13 | Sukemai1 | CSSL.2A.03 | UNL396 |
| 14 | Huaimai30 | CSSL.2A.04 | UNL189 |
| 15 | Zhenmai8 | CSSL.2A.05 | UNL211 |
| 16 | Zhenmai5 | CSSL.2A.06 | UNL576 |
| 17 | Zhenmai4 | CSSL.2A.07 | TAM 113 |
| 18 | Zhenmai168 | CSSL.2B.01 | UNL6 |
| 19 | Zhenmai12 | CSSL.2B.02 | UNL10 |
| 20 | Zhenmai11 | CSSL.2B.03 | UNL611 |
| 21 | Zhenmai10 | CSSL.2B.04 | UNL619 |
| 22 | Zhenmai09196 | CSSL.2B.05 | UNL337 |
| 23 | Zhenmai9523 | CSSL.2B.06 | GALLAGHER |
| 24 | Yangmai25 | CSSL.3A.01 | UNL203 |
| 25 | Yangmai20 | CSSL.3A.02 | UNL88 |
| 26 | Yangmai16 | CSSL.3A.03 | TAM 114 |
| 27 | Yangmai158 | CSSL.3A.04 | UNL575 |
| 28 | Yangfumai8 | CSSL.3A.05 | UNL135 |
| 29 | Yangfumai6 | CSSL.3B.01 | UNL659 |
| 30 | Yangfumai5 | CSSL.3B.02 | Robidoux |
| 31 | Sumai553 | CSSL.3B.03 | SY Wolf |
| 32 | P14 | CSSL.3B.04 | UNL539 |
| 33 | P124 | CSSL.3B.05 | UNL357 |
| 34 | CP20-39-11-2 | CSSL.4A.01 | UNL231 |
| 35 | CP20-39-11-1 | CSSL.4A.02 | UNL30 |
| 36 | CP03-28-1-1 | CSSL.4A.03 | UNL371 |
| 37 | CP02-9-4-8-2 | CSSL.4A.04 | UNL79 |
| 38 | CP02-8-5-6-1 | CSSL.4A.05 | UNL606 |
| 39 | CP02-8-5-5-21 | CSSL.4B.01 | UNL546 |
| 40 | CP02-63-13-1 | CSSL.4B.02 | UNL93 |
| 41 | CP02-62-1-2-2-3F10 | CSSL.4B.03 | Ruth |
| 42 | CP01-39-3-2-4 | CSSL.5A.01 | UNL464 |
| 43 | CP01-39-3-204 | CSSL.5A.02 | UNL437 |
| 44 | CP01-39-17-1-3 | CSSL.5A.03 | UNL515 |
| 45 | Sumai8 | CSSL.5A.04 | UNL268 |
| 46 | Sumai3 | CSSL.5A.05 | UNL627 |
| 47 | Shengxuan6 | CSSL.5A.06 | UNL615 |
| 48 | Ningzimai1 | CSSL.5A.07 | WB CEDAR |
| 49 | Ningmaizi1019 | CSSL.5A.08 | UNL623 |
| 50 | Ningmai26 | CSSL.5B.01 | UNL421 |
| 51 | Ningmai13 | CSSL.5B.02 | SY Wolf |
| 52 | Nannong06Y86 | CSSL.5B.03 | UNL1 |
| 53 | Huamai6 | CSSL.6A.01 | UNL632 |
| 54 | Guohong9 | CSSL.6A.02 | TAM 304 |
| 55 | N/A | CSSL.6A.03 | UNL301 |
| 56 | N/A | CSSL.6A.04 | UNL297 |
| 57 | N/A | CSSL.6A.05 | UNL249 |
| 58 | N/A | CSSL.6A.06 | TAM 113 |
| 59 | N/A | CSSL.6A.07 | UNL100 |
| 60 | N/A | CSSL.6A.08 | UNL288 |
| 61 | N/A | CSSL.6B.01 | UNL573 |
| 62 | N/A | CSSL.6B.02 | NE14696 |
| 63 | N/A | CSSL.6B.03 | UNL584 |
| 64 | N/A | CSSL.6B.04 | UNL293 |
| 65 | N/A | CSSL.6B.05 | UNL40 |
| 66 | N/A | CSSL.7A.01 | UNL66 |
| 67 | N/A | CSSL.7A.02 | UNL535 |
| 68 | N/A | CSSL.7A.03 | UNL3 |
| 69 | N/A | CSSL.7A.04 | UNL360 |
| 70 | N/A | CSSL.7A.05 | Robidoux |
| 71 | N/A | CSSL.7B.01 | UNL15 |
| 72 | N/A | CSSL.7B.02 | UNL45 |
| 73 | N/A | CSSL.7B.03 | UNL580 |
| 74 | N/A | CSSL.7B.04 | Ruth |
| 75 | N/A | CSSL.7B.05 | UNL112 |
| 76 | N/A | CSSL.7B.06 | UNL343 |
| 77 | N/A | CSSL.7B.07 | UNL13 |
| 78 | N/A | CSSL.1D.01 | UNL477 |
| 79 | N/A | CSSL.1D.02 | UNL128 |
| 80 | N/A | CSSL.1D.03 | Freeman |
| 81 | N/A | CSSL.1D.04 | UNL127 |
| 82 | N/A | CSSL.1D.05 | WB CEDAR |
| 83 | N/A | CSSL.1D.06 | UNL401 |
| 84 | N/A | CSSL.2D.01 | UNL364 |
| 85 | N/A | CSSL.2D.02 | UNL29 |
| 86 | N/A | CSSL.2D.03 | GALLAGHER |
| 87 | N/A | CSSL.2D.04 | TAM 114 |
| 88 | N/A | CSSL.2D.05 | UNL620 |
| 89 | N/A | CSSL.3D.01 | UNL368 |
| 90 | N/A | CSSL.3D.02 | NE16443 |
| 91 | N/A | CSSL.3D.03 | WB GRAINFIELD |
| 92 | N/A | CSSL.3D.04 | UNL72 |
| 93 | N/A | CSSL.4D.01 | UNL78 |
| 94 | N/A | CSSL.4D.02 | UNL41 |
| 95 | N/A | CSSL.4D.03 | UNL35 |
| 96 | N/A | CSSL.5D.01 | UNL398 |
| 97 | N/A | CSSL.5D.02 | UNL404 |
| 98 | N/A | CSSL.5D.03 | WB CEDAR |
| 99 | N/A | CSSL.5D.04 | UNL527 |
| 100 | N/A | CSSL.5D.05 | UNL262 |
| 101 | N/A | CSSL.6D.01 | N/A |
| 102 | N/A | CSSL.6D.02 | N/A |
| 103 | N/A | CSSL.6D.03 | N/A |
| 104 | N/A | CSSL.6D.04 | N/A |
| 105 | N/A | CSSL.7D.01 | N/A |
| 106 | N/A | CSSL.7D.02 | N/A |
| 107 | N/A | CSSL.7D.03 | N/A |
| 108 | N/A | CSSL.7D.04 | N/A |
| 109 | N/A | CSSL.7D.05 | N/A |

**Table S2.** The number of plot-based images obtained from drone-collected image series at different growth stages in the trilateral experimental sites.

|  | **Tillering** | **Jointing** | **B&H** | **Flowering** | **Filling** | **Ripening** | **SUM** |
| --- | --- | --- | --- | --- | --- | --- | --- |
| CN | 716 | 758 | 758 | 505 | 800 | 674 | 4212 |
| UK | 326 | 346 | 346 | 230 | 365 | 307 | 1920 |
| US | 173 | 184 | 184 | 122 | 194 | 163 | 1020 |

**Table S3.** Manually scored flowering days in the US field experiment in the 2018/2019 season.

| **Plot ID** | **Plot Range** | **Plot Column** | **Check** | **Flowering dates (in dd/mm/yyyy format)** |
| --- | --- | --- | --- | --- |
| 1 | 1 | 5 | 0 | 23/5/2019 |
| 2 | 1 | 6 | 0 | 23/5/2019 |
| 3 | 1 | 7 | 0 | 5/6/2019 |
| 4 | 1 | 8 | 8 | 24/5/2019 |
| 5 | 1 | 9 | 0 | 13/5/2019 |
| 6 | 1 | 10 | 1 | 23/5/2019 |
| 7 | 1 | 11 | 0 | 18/5/2019 |
| 8 | 1 | 12 | 4 | 23/5/2019 |
| 9 | 1 | 13 | 0 | 2/6/2019 |
| 10 | 1 | 14 | 0 | 24/5/2019 |
| 11 | 2 | 5 | 0 | 24/5/2019 |
| 12 | 2 | 6 | 0 | 23/5/2019 |
| 13 | 2 | 7 | 0 | 24/5/2019 |
| 14 | 2 | 8 | 0 | 24/5/2019 |
| 15 | 2 | 9 | 0 | 24/5/2019 |
| 16 | 2 | 10 | 0 | 23/5/2019 |
| 17 | 2 | 11 | 3 | 24/5/2019 |
| 18 | 2 | 12 | 0 | 23/5/2019 |
| 19 | 2 | 13 | 0 | 23/5/2019 |
| 20 | 2 | 14 | 0 | 23/5/2019 |
| 21 | 3 | 5 | 0 | 25/5/2019 |
| 22 | 3 | 6 | 0 | 24/5/2019 |
| 23 | 3 | 7 | 10 | 26/5/2019 |
| 24 | 3 | 8 | 0 | 24/5/2019 |
| 25 | 3 | 9 | 0 | 24/5/2019 |
| 26 | 3 | 10 | 2 | 24/5/2019 |
| 27 | 3 | 11 | 0 | 26/5/2019 |
| 28 | 3 | 12 | 0 | 23/5/2019 |
| 29 | 3 | 13 | 0 | 24/5/2019 |
| 30 | 3 | 14 | 6 | 28/5/2019 |
| 31 | 4 | 5 | 9 | 25/5/2019 |
| 32 | 4 | 6 | 0 | 25/5/2019 |
| 33 | 4 | 7 | 0 | 25/5/2019 |
| 34 | 4 | 8 | 0 | 25/5/2019 |
| 35 | 4 | 9 | 0 | 23/5/2019 |
| 36 | 4 | 10 | 0 | 25/5/2019 |
| 37 | 4 | 11 | 0 | 22/5/2019 |
| 38 | 4 | 12 | 0 | 26/5/2019 |
| 39 | 4 | 13 | 0 | 23/5/2019 |
| 40 | 4 | 14 | 0 | 23/5/2019 |
| 41 | 5 | 5 | 5 | 25/5/2019 |
| 42 | 5 | 6 | 0 | 23/5/2019 |
| 43 | 5 | 7 | 0 | 25/5/2019 |
| 44 | 5 | 8 | 0 | 25/5/2019 |
| 45 | 5 | 9 | 0 | 25/5/2019 |
| 46 | 5 | 10 | 0 | 24/5/2019 |
| 47 | 5 | 11 | 0 | 22/5/2019 |
| 48 | 5 | 12 | 7 | 24/5/2019 |
| 49 | 5 | 13 | 0 | 4/6/2019 |
| 50 | 5 | 14 | 0 | 23/5/2019 |
| 51 | 6 | 5 | 9 | 25/5/2019 |
| 52 | 6 | 6 | 0 | 25/5/2019 |
| 53 | 6 | 7 | 0 | 25/5/2019 |
| 54 | 6 | 8 | 1 | 24/5/2019 |
| 55 | 6 | 9 | 0 | 24/5/2019 |
| 56 | 6 | 10 | 0 | 24/5/2019 |
| 57 | 6 | 11 | 0 | 24/5/2019 |
| 58 | 6 | 12 | 3 | 25/5/2019 |
| 59 | 6 | 13 | 0 | 24/5/2019 |
| 60 | 6 | 14 | 0 | 25/5/2019 |
| 61 | 7 | 5 | 0 | 24/5/2019 |
| 62 | 7 | 6 | 0 | 26/5/2019 |
| 63 | 7 | 7 | 0 | 25/5/2019 |
| 64 | 7 | 8 | 0 | 24/5/2019 |
| 65 | 7 | 9 | 0 | 23/5/2019 |
| 66 | 7 | 10 | 0 | 24/5/2019 |
| 67 | 7 | 11 | 0 | 26/5/2019 |
| 68 | 7 | 12 | 0 | 24/5/2019 |
| 69 | 7 | 13 | 0 | 24/5/2019 |
| 70 | 7 | 14 | 6 | 27/5/2019 |
| 71 | 8 | 5 | 0 | 25/5/2019 |
| 72 | 8 | 6 | 0 | 25/5/2019 |
| 73 | 8 | 7 | 0 | 25/5/2019 |
| 74 | 8 | 8 | 5 | 25/5/2019 |
| 75 | 8 | 9 | 0 | 24/5/2019 |
| 76 | 8 | 10 | 0 | 25/5/2019 |
| 77 | 8 | 11 | 0 | 22/5/2019 |
| 78 | 8 | 12 | 0 | 25/5/2019 |
| 79 | 8 | 13 | 0 | 24/5/2019 |
| 80 | 8 | 14 | 4 | 24/5/2019 |
| 81 | 9 | 5 | 0 | 23/5/2019 |
| 82 | 9 | 6 | 7 | 24/5/2019 |
| 83 | 9 | 7 | 0 | 26/5/2019 |
| 84 | 9 | 8 | 0 | 25/5/2019 |
| 85 | 9 | 9 | 0 | 23/5/2019 |
| 86 | 9 | 10 | 10 | 27/5/2019 |
| 87 | 9 | 11 | 2 | 24/5/2019 |
| 88 | 9 | 12 | 0 | 24/5/2019 |
| 89 | 9 | 13 | 0 | 24/5/2019 |
| 90 | 9 | 14 | 0 | 24/5/2019 |
| 91 | 10 | 5 | 8 | 25/5/2019 |
| 92 | 10 | 6 | 0 | 24/5/2019 |
| 93 | 10 | 7 | 0 | 23/5/2019 |
| 94 | 10 | 8 | 0 | 25/5/2019 |
| 95 | 10 | 9 | 0 | 25/5/2019 |
| 96 | 10 | 10 | 0 | 26/5/2019 |
| 97 | 10 | 11 | 0 | 25/5/2019 |
| 98 | 10 | 12 | 0 | 25/5/2019 |
| 99 | 10 | 13 | 0 | 25/5/2019 |
| 100 | 10 | 14 | 0 | 24/5/2019 |
| 101 | 11 | 5 | 0 | 23/5/2019 |
| 102 | 11 | 6 | 0 | 26/5/2019 |
| 103 | 11 | 7 | 0 | 25/5/2019 |
| 104 | 11 | 8 | 0 | 25/5/2019 |
| 105 | 11 | 9 | 3 | 25/5/2019 |
| 106 | 11 | 10 | 0 | 25/5/2019 |
| 107 | 11 | 11 | 0 | 24/5/2019 |
| 108 | 11 | 12 | 0 | 24/5/2019 |
| 109 | 11 | 13 | 6 | 26/5/2019 |
| 110 | 11 | 14 | 0 | 25/5/2019 |
| 111 | 12 | 5 | 0 | 25/5/2019 |
| 112 | 12 | 6 | 0 | 24/5/2019 |
| 113 | 12 | 7 | 0 | 24/5/2019 |
| 114 | 12 | 8 | 0 | 24/5/2019 |
| 115 | 12 | 9 | 0 | 24/5/2019 |
| 116 | 12 | 10 | 0 | 24/5/2019 |
| 117 | 12 | 11 | 4 | 25/5/2019 |
| 118 | 12 | 12 | 1 | 29/5/2019 |
| 119 | 12 | 13 | 0 | 9/6/2019 |

**Table S4.** Manually scored flowering days in the UK field experiment in the 2020/2021 season.

| **Plot ID** | **Plot Column** | **Plot Row** | **Flowering dates (in dd/mm/yyyy format)** |
| --- | --- | --- | --- |
| 1 | 1 | 12 | 29/5/2021 |
| 2 | 1 | 11 | 31/5/2021 |
| 3 | 1 | 10 | 29/5/2021 |
| 4 | 1 | 9 | 29/5/2021 |
| 5 | 1 | 8 | 29/5/2021 |
| 6 | 1 | 7 | 26/5/2021 |
| 7 | 1 | 6 | 26/5/2021 |
| 8 | 1 | 5 | 29/5/2021 |
| 9 | 1 | 4 | 29/5/2021 |
| 10 | 1 | 3 | 31/5/2021 |
| 11 | 1 | 2 | 31/5/2021 |
| 12 | 1 | 1 | 22/5/2021 |
| 13 | 2 | 12 | 20/5/2021 |
| 14 | 2 | 11 | 31/5/2021 |
| 15 | 2 | 10 | 29/5/2021 |
| 16 | 2 | 9 | 31/5/2021 |
| 17 | 2 | 8 | 29/5/2021 |
| 18 | 2 | 7 | 31/5/2021 |
| 19 | 2 | 6 | 31/5/2021 |
| 20 | 2 | 5 | 26/5/2021 |
| 21 | 2 | 4 | 29/5/2021 |
| 22 | 2 | 3 | 29/5/2021 |
| 23 | 2 | 2 | 29/5/2021 |
| 24 | 2 | 1 | 26/5/2021 |
| 25 | 3 | 12 | 31/5/2021 |
| 26 | 3 | 11 | 29/5/2021 |
| 27 | 3 | 10 | 31/5/2021 |
| 28 | 3 | 9 | 31/5/2021 |
| 29 | 3 | 8 | 29/5/2021 |
| 30 | 3 | 7 | 31/5/2021 |
| 31 | 3 | 6 | 31/5/2021 |
| 32 | 3 | 5 | 31/5/2021 |
| 33 | 3 | 4 | 29/5/2021 |
| 34 | 3 | 3 | 31/5/2021 |
| 35 | 3 | 2 | 29/5/2021 |
| 36 | 3 | 1 | 31/5/2021 |
| 37 | 4 | 12 | 29/5/2021 |
| 38 | 4 | 11 | 29/5/2021 |
| 39 | 4 | 10 | 29/5/2021 |
| 40 | 4 | 9 | 29/5/2021 |
| 41 | 4 | 8 | 31/5/2021 |
| 42 | 4 | 7 | 29/5/2021 |
| 43 | 4 | 6 | 20/5/2021 |
| 44 | 4 | 5 | 26/5/2021 |
| 45 | 4 | 4 | 31/5/2021 |
| 46 | 4 | 3 | 29/5/2021 |
| 47 | 4 | 2 | 31/5/2021 |
| 48 | 4 | 1 | 3/6/2021 |
| 49 | 5 | 12 | 29/5/2021 |
| 50 | 5 | 11 | 3/6/2021 |
| 51 | 5 | 10 | 29/5/2021 |
| 52 | 5 | 9 | 29/5/2021 |
| 53 | 5 | 8 | 31/5/2021 |
| 54 | 5 | 7 | 31/5/2021 |
| 55 | 5 | 6 | 31/5/2021 |
| 56 | 5 | 5 | 20/5/2021 |
| 57 | 5 | 4 | 26/5/2021 |
| 58 | 5 | 3 | 31/5/2021 |
| 59 | 5 | 2 | 29/5/2021 |
| 60 | 5 | 1 | 29/5/2021 |
| 61 | 6 | 12 | 29/5/2021 |
| 62 | 6 | 11 | 29/5/2021 |
| 63 | 6 | 10 | 31/5/2021 |
| 64 | 6 | 9 | 31/5/2021 |
| 65 | 6 | 8 | 31/5/2021 |
| 66 | 6 | 7 | 31/5/2021 |
| 67 | 6 | 6 | 29/5/2021 |
| 68 | 6 | 5 | 29/5/2021 |
| 69 | 6 | 4 | 31/5/2021 |
| 70 | 6 | 3 | 31/5/2021 |
| 71 | 6 | 2 | 29/5/2021 |
| 72 | 6 | 1 | 29/5/2021 |
| 73 | 7 | 12 | 29/5/2021 |
| 74 | 7 | 11 | 29/5/2021 |
| 75 | 7 | 10 | 31/5/2021 |
| 76 | 7 | 9 | 31/5/2021 |
| 77 | 7 | 8 | 29/5/2021 |
| 78 | 7 | 7 | 29/5/2021 |
| 79 | 7 | 6 | 29/5/2021 |
| 80 | 7 | 5 | 3/6/2021 |
| 81 | 7 | 4 | 29/5/2021 |
| 82 | 7 | 3 | 20/5/2021 |
| 83 | 7 | 2 | 31/5/2021 |
| 84 | 7 | 1 | 7/6/2021 |
| 85 | 8 | 12 | 29/5/2021 |
| 86 | 8 | 11 | 29/5/2021 |
| 87 | 8 | 10 | 29/5/2021 |
| 88 | 8 | 9 | 29/5/2021 |
| 89 | 8 | 8 | 29/5/2021 |
| 90 | 8 | 7 | 29/5/2021 |
| 91 | 8 | 6 | 22/5/2021 |
| 92 | 8 | 5 | 29/5/2021 |
| 93 | 8 | 4 | 5/6/2021 |
| 94 | 8 | 3 | 31/5/2021 |
| 95 | 8 | 2 | 31/5/2021 |
| 96 | 8 | 1 | 29/5/2021 |
| 97 | 9 | 12 | 31/5/2021 |
| 98 | 9 | 11 | 31/5/2021 |
| 99 | 9 | 10 | 5/6/2021 |
| 100 | 9 | 9 | 29/5/2021 |
| 101 | 9 | 8 | 5/6/2021 |
| 102 | 9 | 7 | 5/6/2021 |
| 103 | 9 | 6 | 29/5/2021 |
| 104 | 9 | 5 | 29/5/2021 |
| 105 | 9 | 4 | 29/5/2021 |
| 106 | 9 | 3 | 29/5/2021 |
| 107 | 9 | 2 | 31/5/2021 |
| 108 | 9 | 1 | 29/5/2021 |
| 109 | 10 | 12 | 31/5/2021 |
| 110 | 10 | 11 | 29/5/2021 |
| 111 | 10 | 10 | 29/5/2021 |
| 112 | 10 | 9 | 31/5/2021 |
| 113 | 10 | 8 | 20/5/2021 |
| 114 | 10 | 7 | 5/6/2021 |
| 115 | 10 | 6 | 29/5/2021 |
| 116 | 10 | 5 | 29/5/2021 |
| 117 | 10 | 4 | 29/5/2021 |
| 118 | 10 | 3 | 29/5/2021 |
| 119 | 10 | 2 | 31/5/2021 |
| 120 | 10 | 1 | 29/5/2021 |
| 121 | 11 | 12 | 26/5/2021 |
| 122 | 11 | 11 | 29/5/2021 |
| 123 | 11 | 10 | 31/5/2021 |
| 124 | 11 | 9 | 29/5/2021 |
| 125 | 11 | 8 | 31/5/2021 |
| 126 | 11 | 7 | 20/5/2021 |
| 127 | 11 | 6 | 29/5/2021 |
| 128 | 11 | 5 | 31/5/2021 |
| 129 | 11 | 4 | 31/5/2021 |
| 130 | 11 | 3 | 29/5/2021 |
| 131 | 11 | 2 | 31/5/2021 |
| 132 | 11 | 1 | 29/5/2021 |
| 133 | 12 | 12 | 31/5/2021 |
| 134 | 12 | 11 | 29/5/2021 |
| 135 | 12 | 10 | 29/5/2021 |
| 136 | 12 | 9 | 29/5/2021 |
| 137 | 12 | 8 | 29/5/2021 |
| 138 | 12 | 7 | 31/5/2021 |
| 139 | 12 | 6 | 31/5/2021 |
| 140 | 12 | 5 | 31/5/2021 |
| 141 | 12 | 4 | 31/5/2021 |
| 142 | 12 | 3 | 26/5/2021 |
| 143 | 12 | 2 | 31/5/2021 |
| 144 | 12 | 1 | 29/5/2021 |
| 145 | 13 | 12 | 31/5/2021 |
| 146 | 13 | 11 | 31/5/2021 |
| 147 | 13 | 10 | 29/5/2021 |
| 148 | 13 | 9 | 29/5/2021 |
| 149 | 13 | 8 | 29/5/2021 |
| 150 | 13 | 7 | 29/5/2021 |
| 151 | 13 | 6 | 29/5/2021 |
| 152 | 13 | 5 | 22/5/2021 |
| 153 | 13 | 4 | 29/5/2021 |
| 154 | 13 | 3 | 29/5/2021 |
| 155 | 13 | 2 | 29/5/2021 |
| 156 | 13 | 1 | 29/5/2021 |
| 157 | 14 | 12 | 29/5/2021 |
| 158 | 14 | 11 | 31/5/2021 |
| 159 | 14 | 10 | 31/5/2021 |
| 160 | 14 | 9 | 29/5/2021 |
| 161 | 14 | 8 | 20/5/2021 |
| 162 | 14 | 7 | 29/5/2021 |
| 163 | 14 | 6 | 29/5/2021 |
| 164 | 14 | 5 | 29/5/2021 |
| 165 | 14 | 4 | 31/5/2021 |
| 166 | 14 | 3 | 31/5/2021 |
| 167 | 14 | 2 | 31/5/2021 |
| 168 | 14 | 1 | 29/5/2021 |
| 169 | 15 | 12 | 31/5/2021 |
| 170 | 15 | 11 | 29/5/2021 |
| 171 | 15 | 10 | 29/5/2021 |
| 172 | 15 | 9 | 29/5/2021 |
| 173 | 15 | 8 | 29/5/2021 |
| 174 | 15 | 7 | 31/5/2021 |
| 175 | 15 | 6 | 29/5/2021 |
| 176 | 15 | 5 | 31/5/2021 |
| 177 | 15 | 4 | 29/5/2021 |
| 178 | 15 | 3 | 31/5/2021 |
| 179 | 15 | 2 | 29/5/2021 |
| 180 | 15 | 1 | 29/5/2021 |
| 181 | 16 | 12 | 31/5/2021 |
| 182 | 16 | 11 | 29/5/2021 |
| 183 | 16 | 10 | 20/5/2021 |
| 184 | 16 | 9 | 31/5/2021 |
| 185 | 16 | 8 | 31/5/2021 |
| 186 | 16 | 7 | 29/5/2021 |
| 187 | 16 | 6 | 31/5/2021 |
| 188 | 16 | 5 | 31/5/2021 |
| 189 | 16 | 4 | 5/6/2021 |
| 190 | 16 | 3 | 31/5/2021 |
| 191 | 16 | 2 | 31/5/2021 |
| 192 | 16 | 1 | 29/5/2021 |
| 193 | 17 | 12 | 3/6/2021 |
| 194 | 17 | 11 | 29/5/2021 |
| 195 | 17 | 10 | 3/6/2021 |
| 196 | 17 | 9 | 29/5/2021 |
| 197 | 17 | 8 | 3/6/2021 |
| 198 | 17 | 7 | 29/5/2021 |
| 199 | 17 | 6 | 29/5/2021 |
| 200 | 17 | 5 | 20/5/2021 |
| 201 | 17 | 4 | 26/5/2021 |
| 202 | 17 | 3 | 5/6/2021 |
| 203 | 17 | 2 | 29/5/2021 |
| 204 | 17 | 1 | 26/5/2021 |
| 205 | 18 | 12 | 3/6/2021 |
| 206 | 18 | 11 | 31/5/2021 |
| 207 | 18 | 10 | 29/5/2021 |
| 208 | 18 | 9 | 29/5/2021 |
| 209 | 18 | 8 | 29/5/2021 |
| 210 | 18 | 7 | 29/5/2021 |
| 211 | 18 | 6 | 29/5/2021 |
| 212 | 18 | 5 | 29/5/2021 |
| 213 | 18 | 4 | 29/5/2021 |
| 214 | 18 | 3 | 5/6/2021 |
| 215 | 18 | 2 | 5/6/2021 |
| 216 | 18 | 1 | 5/6/2021 |
| 217 | 19 | 12 | 5/6/2021 |
| 218 | 19 | 11 | 22/5/2021 |
| 219 | 19 | 10 | 31/5/2021 |
| 220 | 19 | 9 | 31/5/2021 |
| 221 | 19 | 8 | 29/5/2021 |
| 222 | 19 | 7 | 29/5/2021 |
| 223 | 19 | 6 | 26/5/2021 |
| 224 | 19 | 5 | 29/5/2021 |
| 225 | 19 | 4 | 31/5/2021 |
| 226 | 19 | 3 | 29/5/2021 |
| 227 | 19 | 2 | 26/5/2021 |
| 228 | 19 | 1 | 26/5/2021 |
| 229 | 20 | 12 | 29/5/2021 |
| 230 | 20 | 11 | 31/5/2021 |
| 231 | 20 | 10 | 22/5/2021 |
| 232 | 20 | 9 | 5/6/2021 |
| 233 | 20 | 8 | 31/5/2021 |
| 234 | 20 | 7 | 3/6/2021 |
| 235 | 20 | 6 | 29/5/2021 |
| 236 | 20 | 5 | 29/5/2021 |
| 237 | 20 | 4 | 26/5/2021 |
| 238 | 20 | 3 | 29/5/2021 |
| 239 | 20 | 2 | 26/5/2021 |
| 240 | 20 | 1 | 29/5/2021 |

**Table S5.** Manually scored flowering days in the Chinese field experiment in the 2019-20 season.

| **Plot ID** | **Plot position in the experiment** | **Flowering dates (in yyyymmdd format)** |
| --- | --- | --- |
| 1 | A201 | 20200413 |
| 2 | A202 | 20200416 |
| 3 | A203 | 20200416 |
| 4 | A204 | 20200414 |
| 5 | A205 | 20200415 |
| 6 | A206 | 20200416 |
| 7 | A207 | 20200414 |
| 8 | A208 | 20200416 |
| 9 | A209 | 20200417 |
| 10 | A210 | 20200418 |
| 11 | A211 | 20200413 |
| 12 | A212 | 20200414 |
| 13 | A213 | 20200412 |
| 14 | A214 | 20200417 |
| 15 | A215 | 20200415 |
| 16 | A216 | 20200414 |
| 17 | A217 | 20200413 |
| 18 | A218 | 20200413 |
| 19 | A219 | 20200416 |
| 20 | A220 | 20200414 |
| 21 | A221 | 20200414 |
| 22 | A222 | 20200418 |
| 23 | A223 | 20200417 |
| 24 | A224 | 20200414 |
| 25 | A225 | 20200416 |
| 26 | A226 | 20200416 |
| 27 | A227 | 20200416 |
| 28 | A228 | 20200412 |
| 29 | A229 | 20200416 |
| 30 | A230 | 20200416 |
| 31 | A231 | 20200416 |
| 32 | A232 | 20200417 |
| 33 | A233 | 20200416 |
| 34 | A234 | 20200414 |
| 35 | A235 | 20200417 |
| 36 | A236 | 20200418 |
| 37 | A237 | 20200415 |
| 38 | A238 | 20200418 |
| 39 | A239 | 20200419 |
| 40 | A240 | 20200415 |
| 41 | A241 | 20200417 |
| 42 | A242 | 20200416 |
| 43 | A243 | 20200418 |
| 44 | A244 | 20200415 |
| 45 | A245 | 20200418 |
| 46 | A246 | 20200415 |
| 47 | A247 | 20200412 |
| 48 | A248 | 20200416 |
| 49 | A249 | 20200413 |
| 50 | A250 | 20200422 |
| 51 | A251 | 20200414 |
| 52 | A252 | 20200415 |
| 53 | A253 | 20200419 |
| 54 | A254 | 20200413 |
| 55 | B101 | 20200413 |
| 56 | B102 | 20200414 |
| 57 | B103 | 20200418 |
| 58 | B104 | 20200418 |
| 59 | B105 | 20200416 |
| 60 | B106 | 20200422 |
| 61 | B107 | 20200416 |
| 62 | B108 | 20200417 |
| 63 | B109 | 20200417 |
| 64 | B110 | 20200417 |
| 65 | B111 | 20200419 |
| 66 | B112 | 20200415 |
| 67 | B113 | 20200415 |
| 68 | B114 | 20200415 |
| 69 | B115 | 20200416 |
| 70 | B116 | 20200414 |
| 71 | B117 | 20200416 |
| 72 | B118 | 20200417 |
| 73 | B119 | 20200416 |
| 74 | B120 | 20200417 |
| 75 | B121 | 20200416 |
| 76 | B122 | 20200416 |
| 77 | B123 | 20200417 |
| 78 | B124 | 20200416 |
| 79 | B125 | 20200418 |
| 80 | B126 | 20200419 |
| 81 | B127 | 20200415 |
| 82 | B128 | 20200422 |
| 83 | B129 | 20200417 |
| 84 | B130 | 20200416 |
| 85 | B131 | 20200416 |
| 86 | B132 | 20200415 |
| 87 | B133 | 20200418 |
| 88 | B134 | 20200418 |
| 89 | B135 | 20200416 |
| 90 | B136 | 20200418 |
| 91 | B137 | 20200416 |
| 92 | B138 | 20200413 |
| 93 | B139 | 20200418 |
| 94 | B140 | 20200417 |
| 95 | B141 | 20200415 |
| 96 | B142 | 20200415 |
| 97 | B143 | 20200415 |
| 98 | B144 | 20200418 |
| 99 | B145 | 20200418 |
| 100 | B146 | 20200416 |
| 101 | B147 | 20200415 |
| 102 | B148 | 20200416 |
| 103 | B149 | 20200413 |
| 104 | B150 | 20200415 |
| 105 | B151 | 20200417 |
| 106 | B152 | 20200418 |
| 107 | B153 | 20200418 |
| 108 | B154 | 20200416 |
| 109 | C101 | 20200418 |
| 110 | C102 | 20200417 |
| 111 | C103 | 20200416 |
| 112 | C104 | 20200415 |
| 113 | C105 | 20200418 |
| 114 | C106 | 20200415 |
| 115 | C107 | 20200414 |
| 116 | C108 | 20200415 |
| 117 | C109 | 20200414 |
| 118 | C110 | 20200414 |
| 119 | C111 | 20200414 |
| 120 | C112 | 20200417 |
| 121 | C113 | 20200416 |
| 122 | C114 | 20200414 |
| 123 | C115 | 20200418 |
| 124 | C116 | 20200416 |
| 125 | C117 | 20200414 |
| 126 | C118 | 20200416 |
| 127 | C119 | 20200415 |
| 128 | C120 | 20200415 |
| 129 | C121 | 20200416 |
| 130 | C122 | 20200415 |
| 131 | C123 | 20200416 |
| 132 | C124 | 20200416 |
| 133 | C125 | 20200413 |
| 134 | C126 | 20200414 |
| 135 | C127 | 20200416 |
| 136 | C128 | 20200418 |
| 137 | C129 | 20200418 |
| 138 | C130 | 20200414 |
| 139 | C131 | 20200418 |
| 140 | C132 | 20200412 |
| 141 | C133 | 20200415 |
| 142 | C134 | 20200416 |
| 143 | C135 | 20200413 |
| 144 | C136 | 20200422 |
| 145 | C137 | 20200416 |
| 146 | C138 | 20200415 |
| 147 | C139 | 20200418 |
| 148 | C140 | 20200414 |
| 149 | C141 | 20200417 |
| 150 | C142 | 20200415 |
| 151 | C143 | 20200412 |
| 152 | C144 | 20200415 |
| 153 | C145 | 20200416 |
| 154 | C146 | 20200415 |
| 155 | C147 | 20200415 |
| 156 | C148 | 20200413 |
| 157 | C149 | 20200415 |
| 158 | C150 | 20200415 |
| 159 | C151 | 20200416 |
| 160 | C152 | 20200417 |
| 161 | C153 | 20200414 |
| 162 | C154 | 20200414 |

**Table S6.** The performance of GSP-AI model across six key stages and at three trial centres, with or without climatic factors.

| **Trilateral field experiments** | | **Tillering** | **Jointing** | **B&H** | **Flowering** | **Filling** | **Ripening** |
| --- | --- | --- | --- | --- | --- | --- | --- |
| **CN** | Single-modal | 0.91 | 0.84 | 0.78 | 0.78 | 0.92 | 0.96 |
|  | GSP-AI | 0.93 | 0.95 | 0.87 | 0.88 | 0.98 | 0.98 |
| **UK** | Single-modal | 0.86 | 0.80 | 0.78 | 0.79 | 0.8 | 0.92 |
|  | GSP-AI | 0.90 | 0.85 | 0.84 | 0.82 | 0.89 | 0.95 |
| **US** | Single-modal | 0.85 | 0.78 | 0.78 | 0.76 | 0.81 | 0.90 |
|  | GSP-AI | 0.89 | 0.82 | 0.83 | 0.83 | 0.84 | 0.94 |

**Table S7**. The number of raw and augmented images collected by smartphone at different growth stages.

| **Growth stages** | **Tillering** | **Jointing** | **B&H** | **Flowering** | **Filling** | **Ripening** |
| --- | --- | --- | --- | --- | --- | --- |
| **Raw images** | 321 | 317 | 304 | 315 | 324 | 322 |
| **Augmented images** | 1,655 | 1,652 | 1,643 | 1,657 | 1,692 | 1,688 |

# Note S1. Flight parameters applied in trilateral drone phenotyping.

We used Pix4Dcapture and DJI GS PRO flight control software to set up drone aerial imaging. To facilitate the reconstruction of 3D point clouds and 2D orthomosaics of the field experiments, we selected “Double Grid Mission” to plan the drone flights. When planning the drone imaging for the first time, pilots were required to accurately position the drone flight mission with in-field ground control points (GCPs) and other geo-referencing points, which were normally placed in each corner of the field to help geo-reference the experimental area. For more accurate geo-referencing results, we used real-time kinematic (RTK) positioning to record Global Navigation Satellite System (GNSS) information from in-field GCPs and reference points. After the in-field setting, both 3D point clouds and 2D orthomosaics could be calibrated in the image pre-processing.

To facilitate the collection of high-quality canopy images with different image resolutions for training deep learning models, we flew at different altitudes to acquire vision-based canopy information (14 m in China, 20 m in the UK, and 25 m in the US). Detailed flight parameters were set on the screen for the flight mission, including drone speed (< 2 metre per second), camera angle (75-80°), image overlap (forward 80% and side 75%). If the airtime was over 20 minutes (airtime for one battery is around 20-25 minutes), the flight mission was split into multiple missions. Once the flight mission was set, we could press the ‘Take off’ button to perform automated aerial imaging. The setting for DJI GS PRO was similar to Pix4Dcapture.

# Note S2. The calculation of accumulation of daily effective temperature.

Accumulation of daily effective temperature (ADET) is widely used for monitoring crop growth [1], and formulae to calculate ADET are listed below:

$$ET=0 if:T_{Avg}\leq T_{Base} (1)$$

$$ET=T_{Avg}-T_{Base} if:T_{Base}<T_{Avg}<T_{Max} (2)$$

$$ET=T_{Max}-T_{Base} if:T_{Avg}\geq T_{Max} (3)$$

$$ADET= \sum_{i=1}^{n} ET_{i} (4)$$

Where the base temperature (T_BASE_) was set to 3 °C and the maximum effective temperature (T_Max_) was set to 30 °C for the Chinese trial, whereas T_BASE_ was set to 0 °C and T_UP_ was set to 25 °C for the UK and US trials [2]; ET denotes effective temperature, T_Avg_ stands for average temperature within a day, *n* denotes the number of days included in the calculation of ADET.

Accumulated Solar Radiation (ASR), which can be obtained by summing the daily solar radiation, formulae to calculate ASP are below:

$$ASR= \sum_{i=1}^{n} SR_{i} (5)$$

Where $n$ denotes the number of days after sowing (DAS), ${SR}_{i}$ and denotes the solar radiation at the i^th^ DAS.

# Note S3. LSTM for modelling climatic changes.

We utilised LSTM to extract temporal changes from environmental datasets. As a recurrent neural network (RNN), LSTM uses “gate” mechanism to handle sequential input data to determine the degree to which LSTM neural units keep the previous state and memorise the current feature [3] . For example, “Forget gate” controls the output information of the previous time point, determining which information can be transmitted to the current time. The output value is generated by the sigmoid function, where the size of the *f_t* is between 0-1, where 1 stands for completely retained the information and 0 stands for completely discarded the information.

$$f_{t}=\sigma\left( W_{f}\cdot\left[ h_{t-1},x_{t} \right]+b_{f} \right)$$

Where $W_{f}$, $b_{f}$ are learnable weight matrices, $\sigma$ is activation functions, $x_{t}$ is the input of the current time point, and $h_{t-1}$ is the output of the previous time point.

Input gate and memory unit decide what kind of new information is passed to the next neural unit, as well as whether the memory unit shall be updated. Using the sigmoid function to generate the input value $i_{t}$, the tanh function to generate the candidate value $\tilde{C_{t}}$, $i_{t}\cdot\tilde{C_{t}}$ can result in new candidate information, with $f_{t}\cdot C_{t-1}$ to forget unwanted information in the memory unit. The new memory stream information$C_{t}$ can be computed using the formulae below.

$$i_{t}=\sigma\left（ W_{i}\cdot\left[ h_{t-1},x_{t} \right] \right）+b_{i}$$

$$\tilde{C_{t}}=\tanh\left( W_{c}\cdot\left[ h_{t-1},x_{t} \right] \right)+b_{c}$$

$$C_{t}= f_{t}\cdot C_{t-1}+i_{t}\cdot\tilde{C_{t}}$$

Where $W_{i},W_{i}b_{i},b_{c}$ are learnable weight matrices, $\sigma$ and $\tanh$ are activation functions.

In our case, the attention module adjusts the contribution of each hidden feature to the output module by normalising the weight parameters for target climatic factors one-season data for the UK/US trials and multiple-seasons for the Chinese trial using factors such as accumulative growing degree day, ASR and ADET. The attention network consists of a one-layer fully connected network with the Softmax function, yielding weight values α _t_ for each hidden feature vector *ht* that is obtained from the LSTM module. For each time step *t*, the hidden feature after adding the attention module is $h_{t}^{*}$.

$$\alpha_{t}=softmax(W_{a}\cdot h_{t}+b_{a})$$

$$h_{t}^{*}= \alpha_{t} \cdot h_{t}$$

Where $W_{a}$, $b_{a}$ are learnable weight matrices.

# Figure S1. After fine-tuning, the changes in accuracies and loss values of the different models on the training set and validation set.


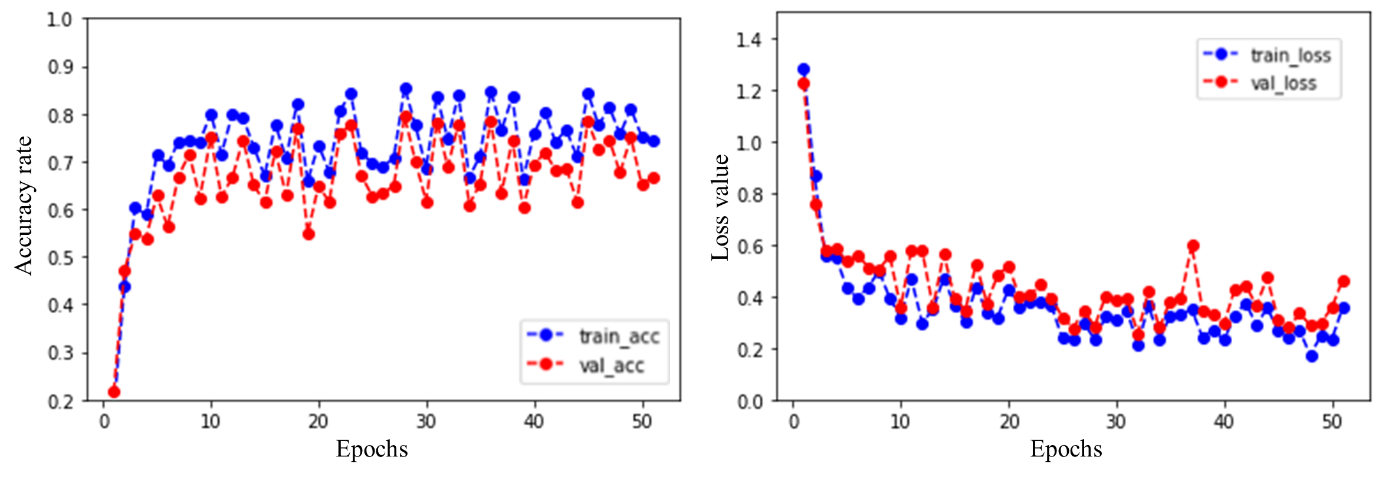


**Figure S3.1** The accuracy and loss values of VGG-16 model


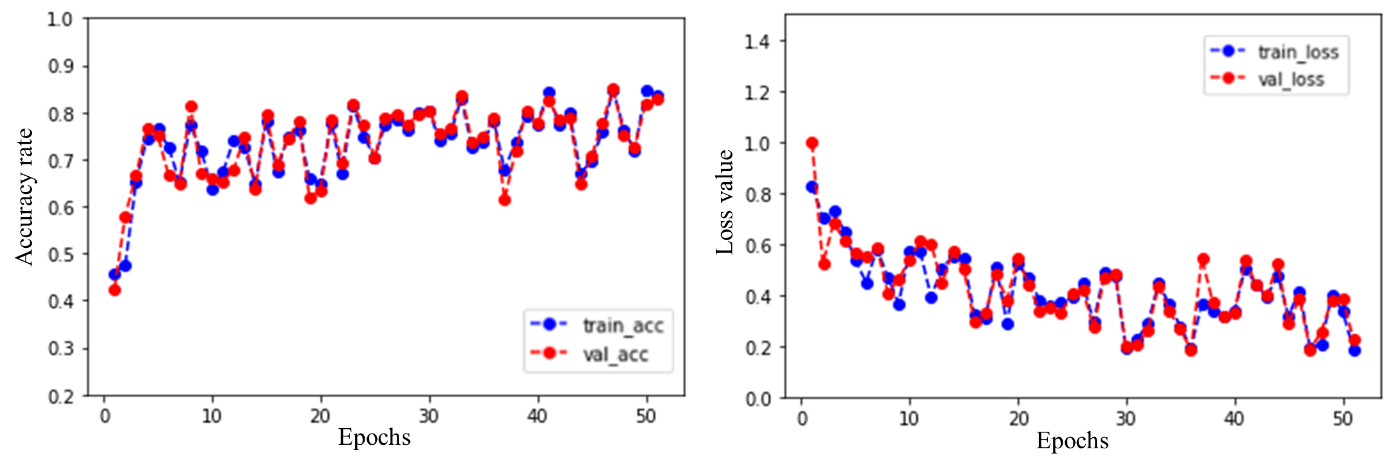


**Figure S3.2** The accuracy and loss values of Inception-v3


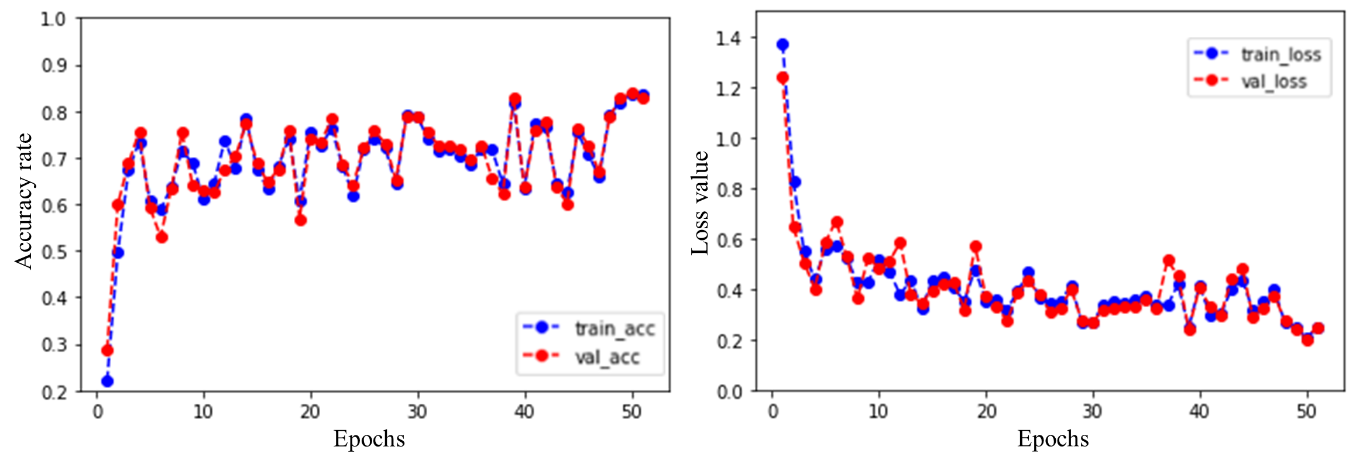


**Figure S3.3** The accuracy and loss values of DenseNet-121


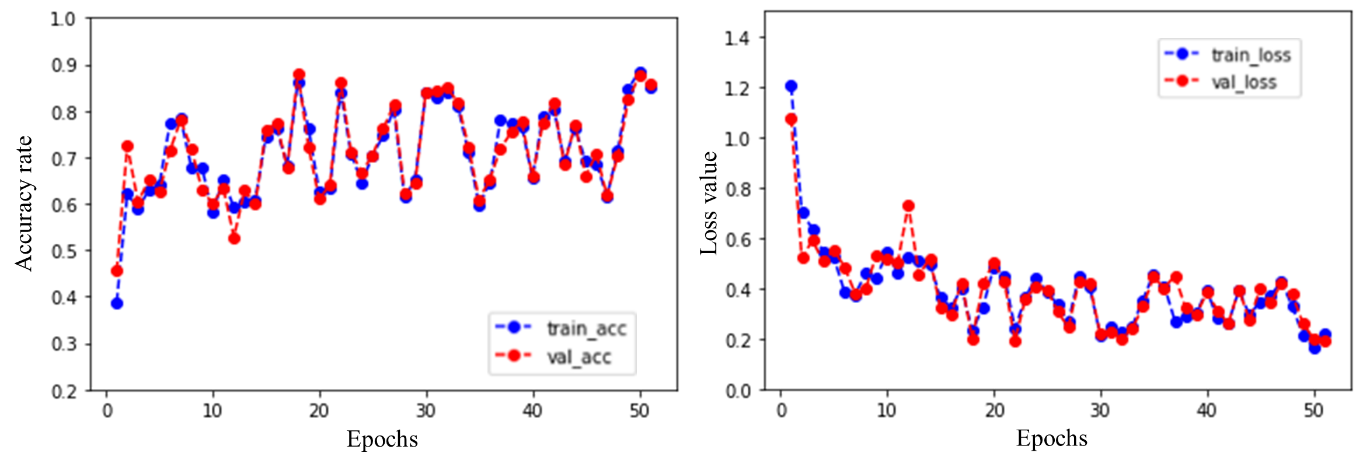


**Figure S3.4** The accuracy and loss values of ResNet-101


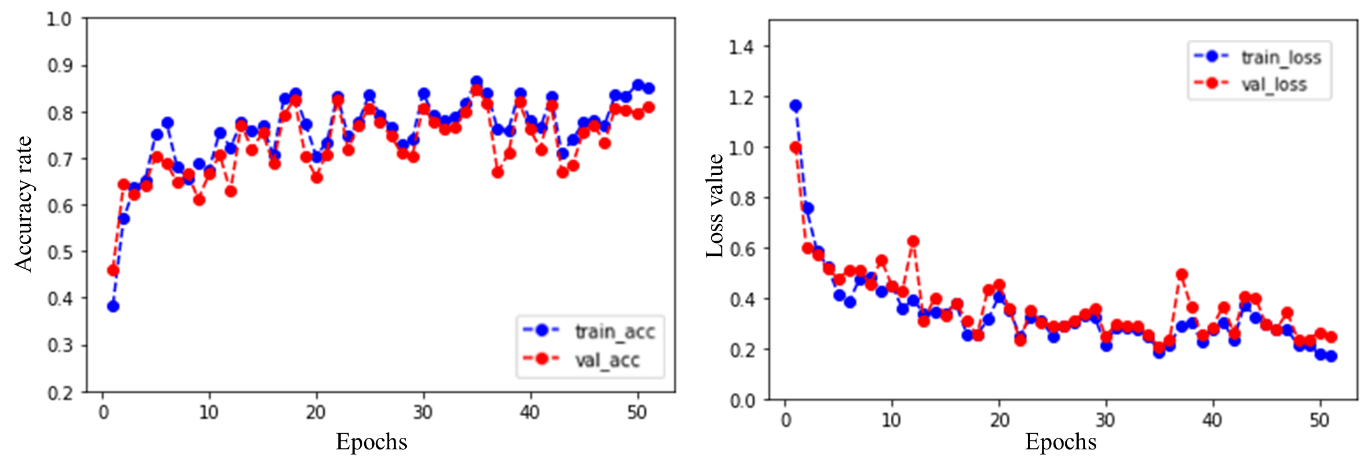


**Figure S3.5** The accuracy and loss values of ResNet-19


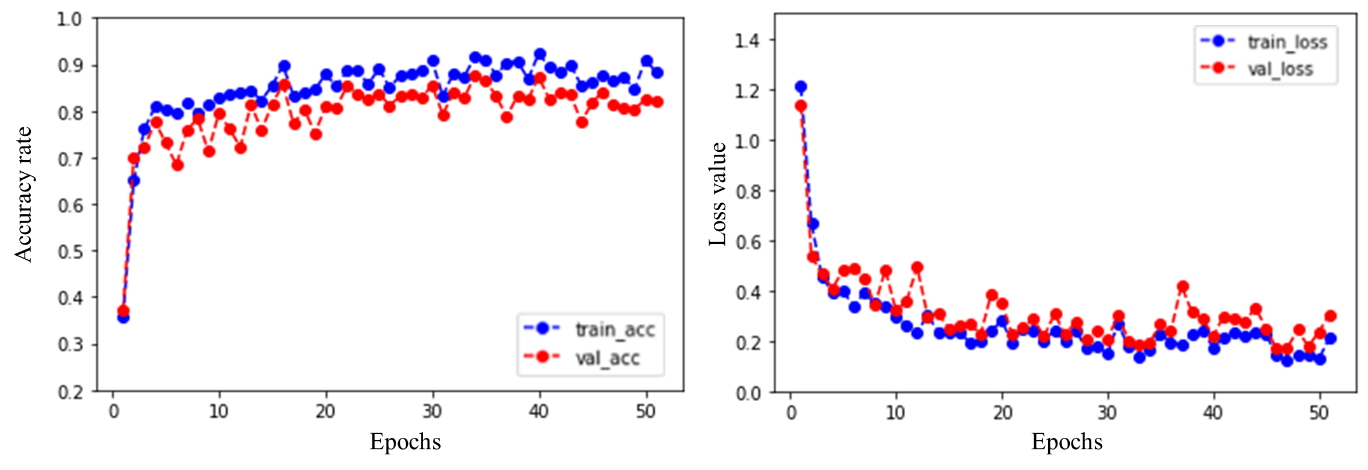


**Figure S3.6** The accuracy and loss value of Res2Net-19


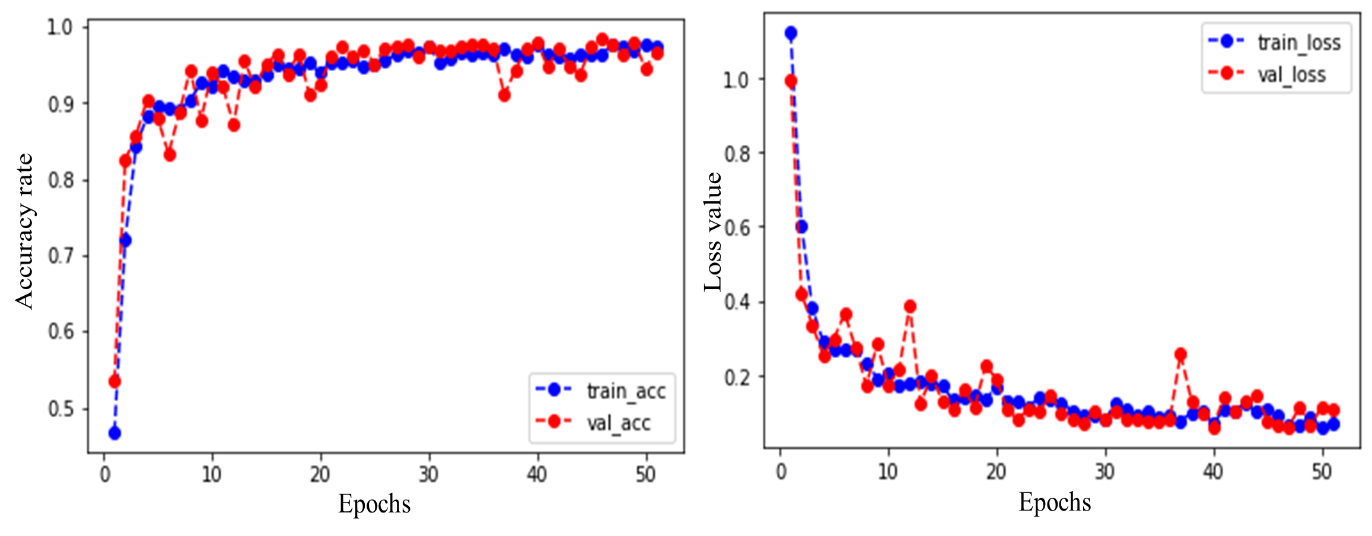


**Figure S3.7** The accuracy and loss value of GSP-AI model

# Figure S2. Key climate factors and their changing patterns in the three field experiments


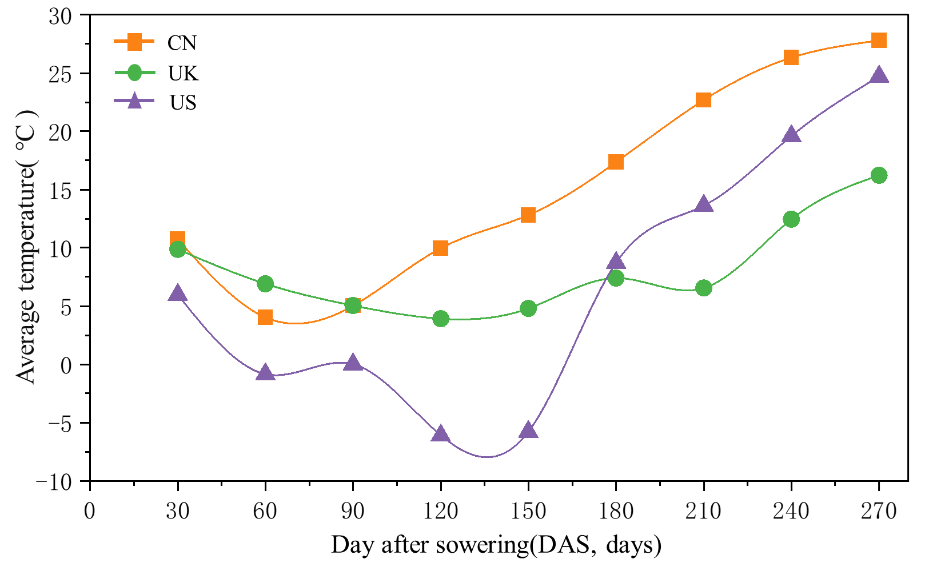


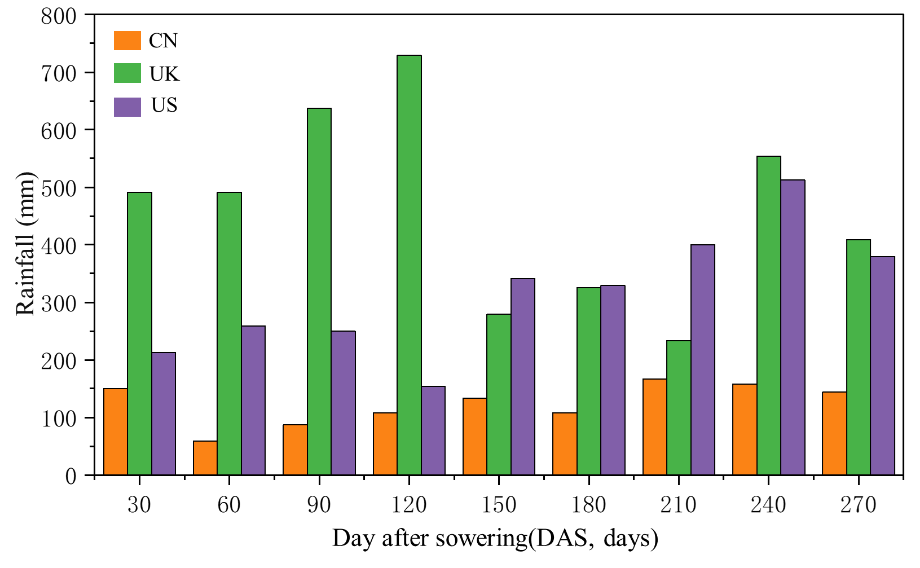


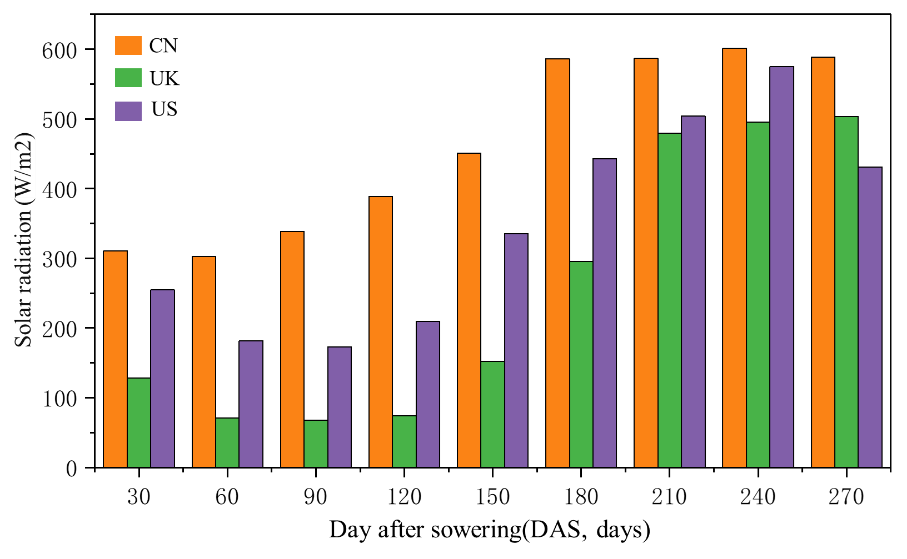


# Figure S3. Correlation analyses between AI-estimated and manually scored flowering days based on smartphone cameras.


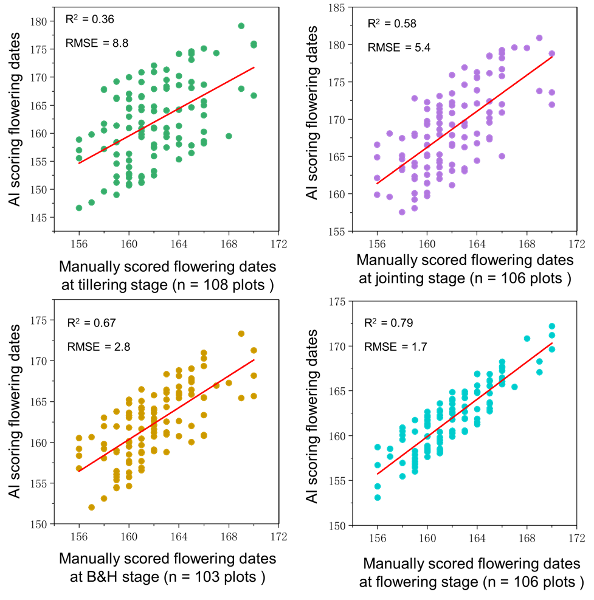


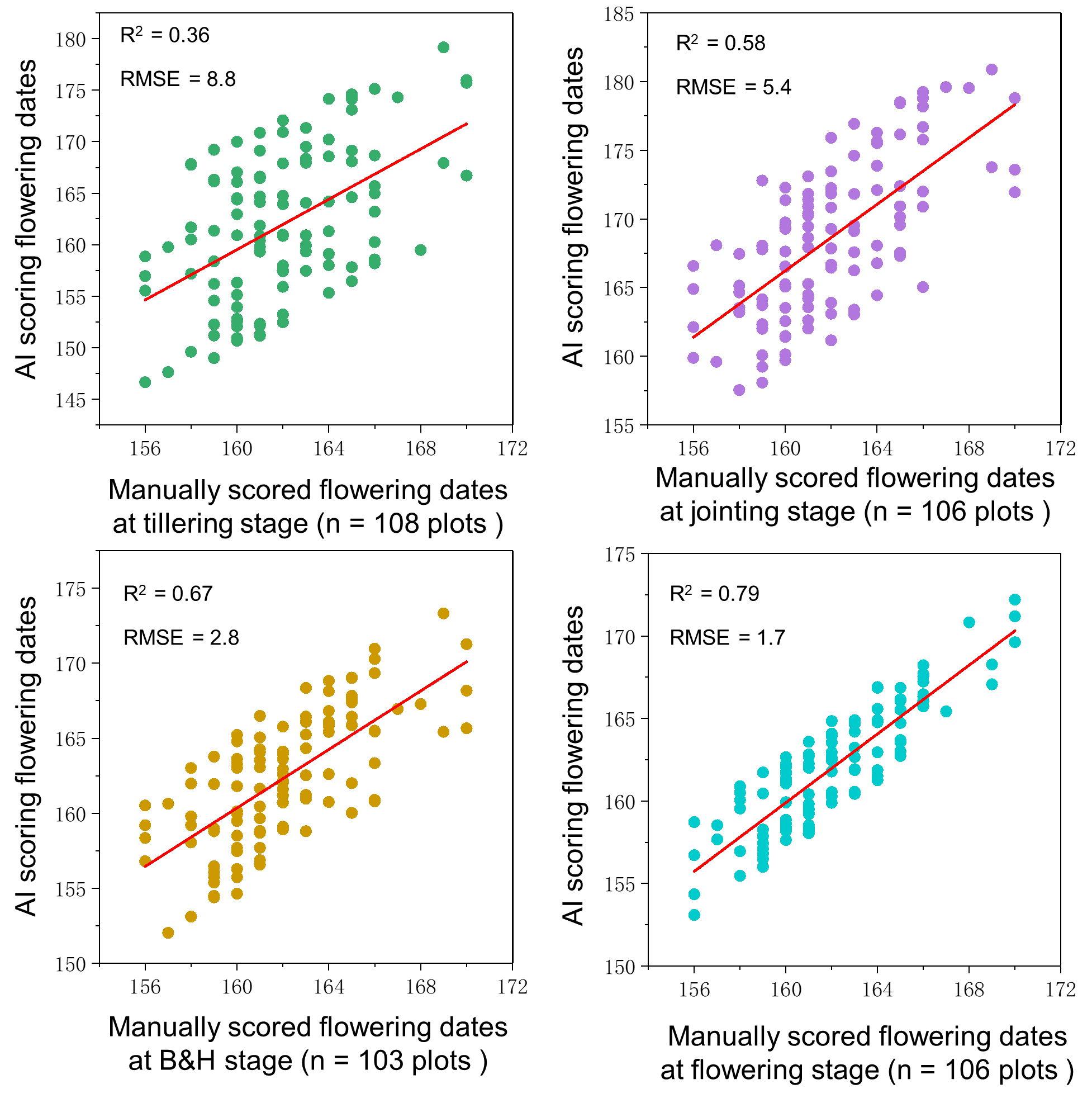


# Data S1. Collected meteorological datasets used in this study

The complete environment factors for the trilateral trials across 2018-2022 growing seasons. The dataset can also be downloaded via the journal or our GitHub repository (<https://github.com/The-Zhou-Lab/GSP-AI/releases>).

# References:

1. Seppelt R, Klotz S, Peiter E, Volk M. Agriculture and food security under a changing climate: An underestimated challenge. iScience [Internet]. The Author(s); 2022;25:105551. Available from: https://doi.org/10.1016/j.isci.2022.105551

2. McMaster GS, White JW, Hunt LA, Jamieson PD, Dhillon SS, Ortiz-Monasterio JI. Simulating the influence of vernalization, photoperiod and optimum temperature on wheat developmental rates. Ann Bot. 2008;102:561–9.

3. Wang Q, Peng RQ, Wang JQ, Li Z, Qu HB. NEWLSTM: An Optimized Long Short-Term Memory Language Model for Sequence Prediction. IEEE Access. IEEE; 2020;8:65395–401.
